# Selected by gene co-expression network and molecular docking analyses, ENMD-2076 is highly effective in glioblastoma-bearing rats

**DOI:** 10.18632/aging.102422

**Published:** 2019-11-09

**Authors:** Sheng Zhong, Yang Bai, Bo Wu, Junliang Ge, Shanshan Jiang, Weihang Li, Xinhui Wang, Junan Ren, Haiyang Xu, Yong Chen, Gang Zhao

**Affiliations:** 1 Department of Neurosurgery, The First Hospital of Jilin University, Changchun, China; 2 Department of Cancer Biology, Dana-Farber Cancer Institute, Boston, MA 02115, USA; 3 Department of Bioinformatics, Harvard Medical School, Boston, MA 02115, USA; 4 Clinical College, Jilin University, Changchun, China; 5 Department of Orthopedics, The First Hospital of Jilin University, Changchun, China; 6 Institute of Zoology, China Academy of Science, Beijing, China; 7 Department of Oncology, The First Hospital of Jilin University, Changchun, China

**Keywords:** bioinformatics, brain science, hub genes, glioblastoma

## Abstract

Background: Glioblastoma is the most common type of malignant brain tumor. Bioinformatics technology and structure biology were effectively and systematically used to identify specific targets in malignant tumors and screen potential drugs.

Results: GBM patients have higher AURKA and KDR mRNA expression compared with normal samples. Then, we identified a small molecular compound, ENMD-2076, could effectively inhibit Aurora kinase A and VEGFR-2 (encoded by KDR) activities. ENMD-2076 is predicted without toxic properties and also has absorption and gratifying brain/blood barrier penetration ability. Further results demonstrated that ENMD-2076 could significantly inhibit GBM cell lines proliferation and vitality, it also suppressed GBM cells migration and invasion. ENMD-2076 induced glioblastoma cell cycle arrest in G2-M phase and apoptosis by inhibiting PI3K/AKT/mTOR signaling pathways. Additionally, ENMD-2076 prolonged the median survival time of tumor-bearing rats and restrained growth rate of tumor volume *in vivo*.

Conclusions: Our findings reveal that ENMD-2076 is a promising drug in dealing with glioblastoma and have a perspective application.

Methods: We show that AURKA and KDR genes are hub driver genes in glioblastoma with bioinformatics technology including WGCNA analysis, PPI network, GO, KEGG analysis and GSEA analysis. After identifying a compound via virtual screening analysis, further experiments were carried out to examine the anti-glioblastoma activities of the compound *in vivo* and *in vitro*.

## INTRODUCTION

Glioblastoma is the most common type of malignant brain tumor characterized by poor prognosis and direct repercussions on cognitive function. The annual incidence of glioblastoma is estimated 5.26 per 100,000 population [1]. According to the 2016 World Health Organization (WHO) classification of central nervous system tumors, glioblastomas can be divided into a series molecular subtype based on IDH (isocitrate dehydrogenase) mutation, EGFR (epidermal growth factor receptor) status, alterations in TP53, methylation of MGMT, 1p19q co-deletion and etc [2, 3]. The standard therapies in dealing with glioblastoma include surgical resection, chemotherapy and radiotherapy [4, 5]. With standard therapies, the median survival for adult patients with IDH-mutant glioblastoma is 27–31 months while IDH-wildtype glioblastoma is only 11–15 months [6]. Owning to overall dismal prognosis and poor life quality of glioblastoma with standard treatment, it is urgent to explore novel treatment and screen effective medication for glioblastoma under better understanding of genetic and molecular biology.

In recent years, bioinformatics and microarray technology are widely employed to analyze molecular and genetic mechanism of malignant tumors [7]. These methods identify core driving genes and abnormal regulation pathways of disease by applying corresponding bioinformatics algorithms. Bioinformatics analysis help researchers reveal therapeutic molecular targets, theoretic basis of tumor onset, progression, or metastasis in a systematical, accurate and effective manner. Weighted gene co-expression network analysis (WGCNA) is an emerging systems biology method to process gene expression data and explore network alterations [8–10]. Assisted by WGCNA, researchers could explore the underlying mechanism among highly correlated genes and discover novel diagnostic biomarkers or therapeutic targets from disease associated genes cluster. After elucidating and validating fundamental molecular biological process, investigators could further perform molecular docking to screen small molecular drugs which could combine with selected targets [11]. Virtual screening and molecular docking is a widely applied method in rational drug design and medicinal chemistry [11, 12]. It not only provides binding affinity between protein and ligand at the atomic level, but also calculates a series of pharmacological properties of specific ligands [13, 14]. Therefore, bioinformatics combined with virtual screen analysis were employed by us to accelerate glioblastoma drug discovery.

**Figure 1:**
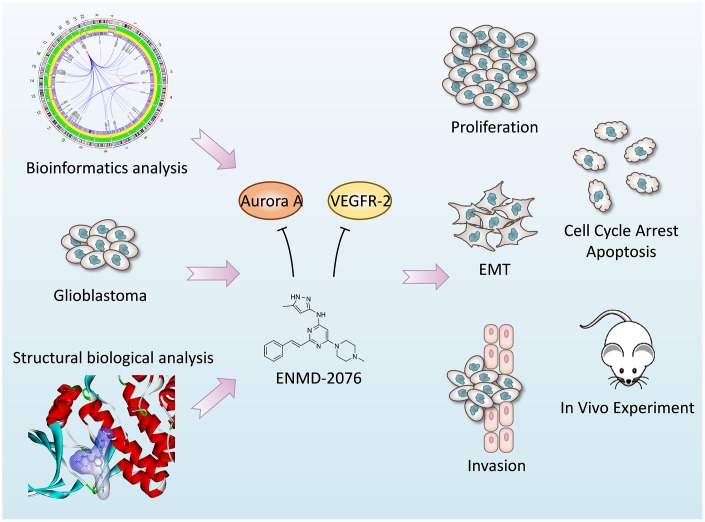
**The diagram of this study.**

In current study, microarray datasets GSE50161 has been analyzed to identify potential therapeutic targets and key pathways. Gene Ontology (GO), Kyoto Encyclopedia of Genes and Genomes (KEGG) analyses were carried out to discover molecular function and abnormal regulated pathways. Clinical patients’ datasets were calculated to verify prognostic impact of *AURKA* and *KDR* expression. Next, we applied molecular docking method to identify ENMD-2076, which is a novel small molecule inhibitor of Aurora kinase and tyrosine kinase, as a potential anti-glioblastoma drug [15]. Finally, *in vitro* and *in vivo* experiments were performed to verify the therapeutic effect of ENMD-2076 on glioblastoma as well as to elucidate molecular biological mechanism. The diagram of this study is displayed in Figure 1. This study provides a novel medication candidate for glioblastoma treatment.

## RESULTS

### Construction of weighted gene co-expression networks and key module identification

**Figure 2:**
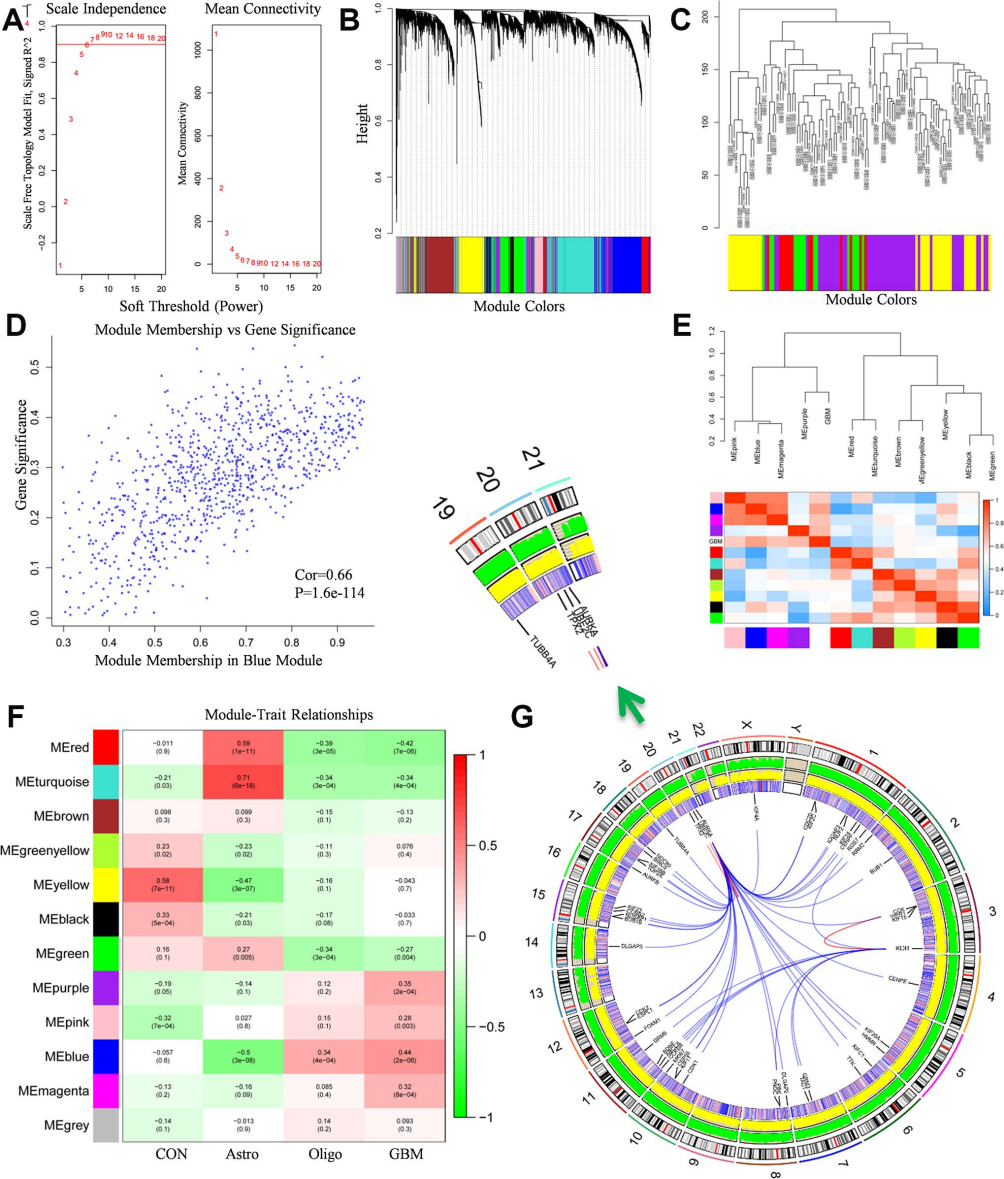
**WGCNA analysis results.** (**A**) The soft-thresholding powers selection of WGCNA analysis. The left panel showed the scale-free fit index (y-axis) as a function of soft-thresholding power (x-axis). The red line represented the y-axis value. The right panel showed the mean connectivity (y-axis) as a function of soft-thresholding powers (x-axis). (**B**) Module assignment and cluster dendrogram. Highly interconnected groups of genes were clustered. Modules were represented by different colors in the horizontal bar. 12 modules were identified with the hierarchical clustering tree analysis. (**C**) Cluster dendrogram of samples in GSE50161 to detect outliers. The dendrogram branches represented the clustered samples. (**D**) The correlation between gene significance and module membership in the blue module. The y-axis represented the gene significance in the blue module. The x-axis represented the module membership in the blue module. (**E**) The heat map of adjacencies in the eigengene network. The adjacency of corresponded modules was represented by column and row square color. High adjacency was represented with red while low adjacency was represented with blue. (**F**) The module-trait relationship heat map. The column represented clinical features. The first column was control samples. The second column was ependymoma. The third column was medulloblastoma. The fourth column was glioblastoma. The row represented the module eigengene. The first line in every square was the corresponding correlation. The second line in every line was the p-value. The left side of heat map indicated the module name. The right side of heat map indicated the colors of correlation (green represented negatively correlated, red represented positively correlated). (**G**) The Circos plot of GBM genome showed chromosome structure, gene expression level and gene encoding protein interaction relationship. The chromosome model was the most outer layer. The green and yellow track represented average gene expression level of normal brain tissue and glioblastoma in GSE50161, respectively. The inner track which is consisted of red and blue stripe stand for the p-value in comparison of normal brain tissue and glioblastoma, high p-value was represented with red, vice versa. The most inner track denoted gene encoding protein interaction relationship.

The microarray dataset GSE50161 were applied into this study, gene expression profiles of normal brain samples and surgical tumor samples (n=130) were normalized and generated. Firstly, optimal soft threshold power for WGCNA was calculated. The scale free topology model fit index was almost 0.9 when the soft threshold power was 7 (Figure 2A). Meanwhile, the mean connectivity was almost 0 when the soft threshold power was 7. The results manifested that the networks were scale-free when the soft threshold power was 7. Therefore, power 7 was selected as soft threshold power.

Based on the co-expression relationships, we then performed the hierarchical clustering tree analysis. Results showed 12 gene modules were generated and identified (Figure 2B). The modules were labeled by 12 different colors (red, turquoise, brown, green yellow, yellow, black, green, purple, pink, blue, magenta, grey). Dendrogram branches indicated the samples in each subgroup were highly heterogeneous (Figure 2C). Therefore, it’s necessary to perform WGCNA to identify hub driver genes of each subgroup. Module eigengene adjacency was subsequently calculated to evaluate the entire modules interaction (Figure 2E). Based on the heat map, every module exhibited independent validation to each other module. Red colors and blue colors stand for the different co-expression interconnectedness.

After obtaining WGCNA network data, module-trait relationship was also evaluated. The relationships between every module and normal brain samples, ependymoma samples, medulloblastoma samples, glioblastoma samples were evaluated (Figure 2F). In the column of GBM, the relationship between blue module and GBM (r=0.44, p=2e-6) was significantly higher than other groups, which implied that the blue module was highly correlated with GBM, the genes in blue module play pivotal roles in the pathogenesis and oncogenesis of GBM. Besides, Module membership (MM) vs Gene Significance (GS) scatterplot of blue module was also created (Figure 2D). Results indicated that MM was highly correlated with GS in blue module (Cor=0.66, *p*=1.6e-144). As a result, blue module was identified as a key module accounting for glioblastoma oncogenesis and subsequent analysis was conducted based on blue module genes.

### Identification of hub genes and PPI (protein to protein interaction) network construction

**Figure 3:**
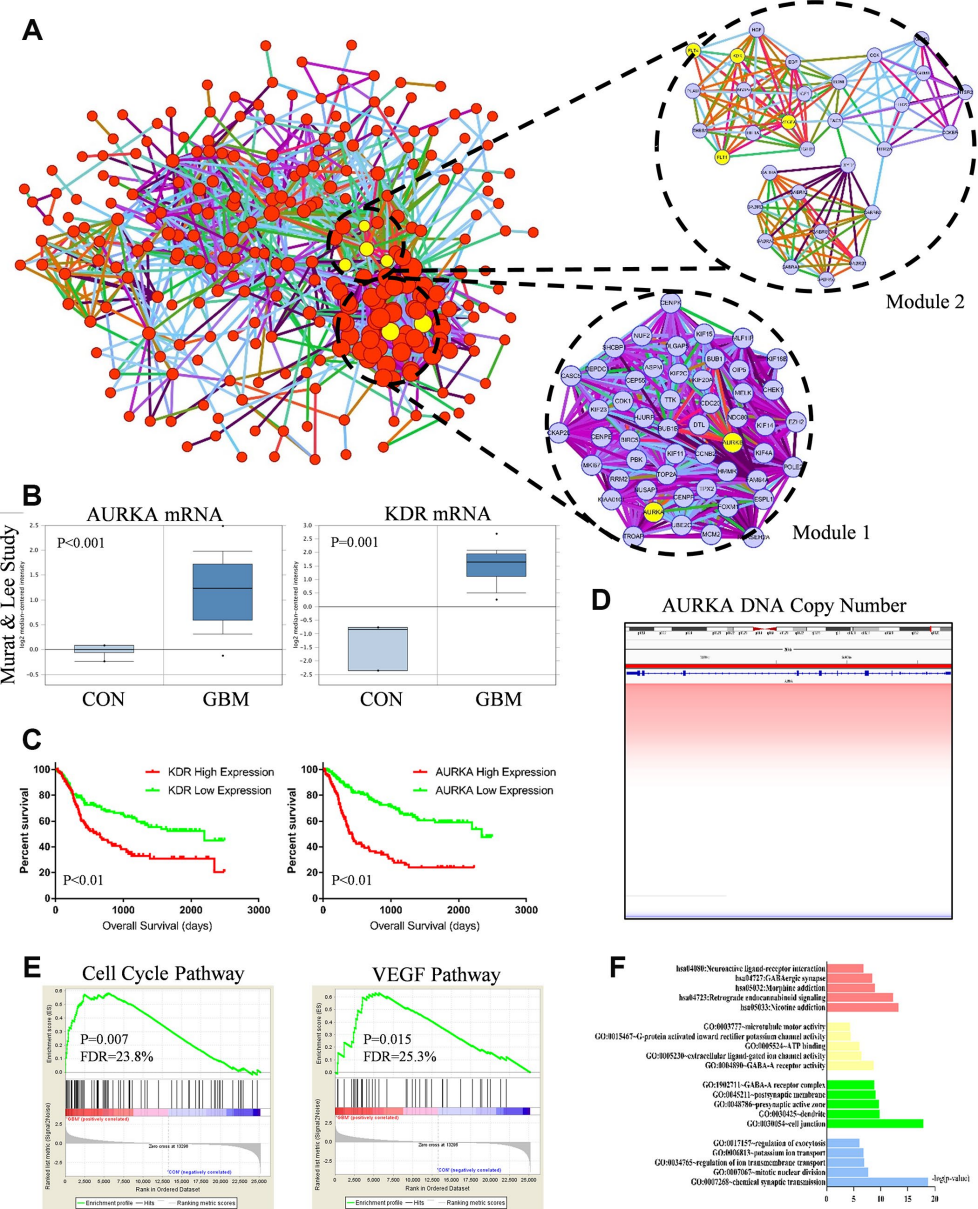
(**A**) Protein-protein interaction (PPI) network of genes in the blue module. Every edge represented the interaction between two genes. Module 1 and module 2 were the top 2 modules from the PPI network. Yellow nodes noted hub nodes. (**B**) Based on the Murat and Lee study, *AURKA* and *KDR* mRNA expression were significant up-regulated in GBM tissues compared with normal brain tissue (p<0.01). (**C**) Kaplan-Meier estimated overall survival (OS) days in GBM patients. Left panel showed low-expressed *KDR* had significantly longer OS days (p=0.01). Right panel showed low-expressed *AURKA* had significantly longer OS days (p=0.01). (**D**) DNA copy number of *AURKA*. (**E**) Gene set enrichment analysis (GSEA) showed that cell cycle pathway (p=0.007, FDR=23.8%) and VEGF pathway (P=0.015, FDR=25.3%) were abnormally regulated in GBM tissues compared with normal brain tissue. (**F**) Functional and pathway enrichment analysis of genes in blue module.

PPI analysis results and gene expression were combined to visualize data in the Circos plot (Figure 2G). Results showed that *AURKA*, *KDR* had most links with other proteins. It indicated that *AURKA* and *KDR* play crucial roles in the pathogenesis and oncogenesis of GBM. Besides, the total genes in blue module were analyzed by PPI network and displayed in Cytoscape. A gene was represented as a node in the PPI network, while the interaction between genes was represented as the edge (Figure 3A). 267 nodes and 2207 edges were acquired. Hub nodes were selected out with degrees≥55. As a result, 21 nodes were recognized as hub genes including *AURKA*, *AURKB*, *KDR*, *TOP2A*, etc (Supplementary Table 1). Among the hub nodes, *AURKA* possessed 56 node degrees and *KDR* possessed 19 node degrees. Subsequently, 2 significant modules were obtained after MCODE analysis (Figure 3A). As shown in Figure 3A, module 1 contained 52 nodes and 1232 edges. Module 2 contained 31 nodes and 157 edges. The detail information of the 2 gene modules was also displayed in the Supplementary Table 2. The genes in the 2 modules were highly correlated with mitosis, cell cycle, protein translation, angiogenesis.

At the same time, we tested the gene expression levels between GBM and normal samples. The results of gene expression levels were shown in the heat map (Supplementary Figure 1G). We found the *AURKA* and *KDR* expression were much higher in GBM samples compared with normal samples. Additionally, *AURKA* and *KDR* associated genes including *VEGFA*, *AURKB*, *CDC20*, *UBE2C*, *CDK1*, *BUB1*, *BIRC5*, *TPX2* and etc were also abnormally, which demonstrated that *AURKA* and *KDR* associated pathway were regulated aberrantly.

### Functional and pathway enrichment analysis

GO, KEGG pathway analysis were used to identify further insight of genes in blue module. The results of GO and KEGG analysis were shown in Figure 3F and Table 1. It indicated that 15 cellular functions and 5 signaling pathways were identified to be significant changed. The cellular functions included mitotic nuclear division, cell junction, ATP binding, etc. The signaling pathways included retrograde endo-cannabinoid signaling, neuro-active ligand-receptor interaction, etc. Besides, GSEA analysis was also employed to identify the abnormal regulated pathways in GBM patients. As shown in Figure 3E, cell cycle pathway (p=0.007, FDR=23.8%) and VEGF pathway (P=0.015, FDR=25.3%) were most commonly enriched signaling pathways. GSEA analysis indicated that cell cycle pathway (*AURKA* involved) and VEGF pathway (*KDR* involved) were aberrantly activated in GBM patients, the results were highly consistent with hub gene identification results.

**Table 1:** Functional and pathway enrichment analysis of the genes in blue module.

**Category**	**Term**	**Count**	**%**	**PValue**
GOTERM_BP_DIRECT	GO:0007268~chemical synaptic transmission	36	8.49	1.56E-19
GOTERM_BP_DIRECT	GO:0007067~mitotic nuclear division	23	5.42	1.82E-08
GOTERM_BP_DIRECT	GO:0034765~regulation of ion transmembrane transport	15	3.54	1.01E-07
GOTERM_BP_DIRECT	GO:0006813~potassium ion transport	13	3.07	1.59E-07
GOTERM_BP_DIRECT	GO:0017157~regulation of exocytosis	8	1.89	8.76E-07
GOTERM_CC_DIRECT	GO:0030054~cell junction	47	11.08	1.17E-18
GOTERM_CC_DIRECT	GO:0030425~dendrite	30	7.08	1.51E-10
GOTERM_CC_DIRECT	GO:0048786~presynaptic active zone	11	2.59	2.26E-10
GOTERM_CC_DIRECT	GO:0045211~postsynaptic membrane	23	5.42	8.23E-10
GOTERM_CC_DIRECT	GO:1902711~GABA-A receptor complex	9	2.12	1.37E-09
GOTERM_MF_DIRECT	GO:0004890~GABA-A receptor activity	9	2.12	2.18E-09
GOTERM_MF_DIRECT	GO:0005230~extracellular ligand-gated ion channel activity	9	2.12	3.10E-07
GOTERM_MF_DIRECT	GO:0005524~ATP binding	61	14.39	7.72E-07
GOTERM_MF_DIRECT	GO:0015467~G-protein activated inward rectifier potassium channel activity	5	1.18	3.65E-05
GOTERM_MF_DIRECT	GO:0003777~microtubule motor activity	10	2.36	4.53E-05
KEGG_PATHWAY	hsa05033:Nicotine addiction	15	3.54	5.62E-14
KEGG_PATHWAY	hsa04723:Retrograde endocannabinoid signaling	20	4.72	4.40E-13
KEGG_PATHWAY	hsa05032:Morphine addiction	16	3.77	1.04E-09
KEGG_PATHWAY	hsa04727:GABAergic synapse	15	3.54	3.89E-09
KEGG_PATHWAY	hsa04080:Neuroactive ligand-receptor interaction	23	5.42	1.61E-07

### Survival curve analysis

Survival curve analysis was performed to discover the relationship between *AURKA*, *KDR* expression and GBM prognosis in the TCGA, CGGA dataset. In the CGGA dataset, GBM patients with low-expressed *AURKA* had significantly favorable overall survival (OS) days compared with high-expression *AURKA* (95% confidence interval (CI), 2.488-5.020; hazard ratio (HR), 3.506; p<0.01) (Figure 3C). GBM patients with low-expressed *KDR* had significantly prolonged OS days compared with GBM patients with high-expression *KDR* (CI, 1.335-2.641; HR, 1.878; p<0.01). These results indicated that GBM patients with high expressed *AURKA* and *KDR* had poor prognosis. In TCGA dataset, the OS difference between high-expression and low-expression *AURKA* was not significant (*p* value>0.05, Supplementary Figure 1D). However, patients with low-expressed *KDR* had significantly longer OS days compared with GBM patients with high-expression *KDR* (p=0.04).

The aberrant *AURKA* and *KDR* mRNA expression were also analyzed with Oncomine to clarify the difference between normal samples and GBM samples. In the Murat and Lee study, the expression of GBM patients had significantly higher expression of *AURKA* and *KDR* mRNA expression compared with normal samples (p<0.01, Figure 3B). In the Sun Study, *AURKA* and *KDR* mRNA expression in GBM samples was both significantly higher than normal samples (p<0.001, Supplementary Figure 1A).

Apart from mRNA analysis, we also investigated *AURKA* and *KDR* DNA copy number and methylation status. In the Beroukhim Study and TCGA Study, DNA copy number of *AURKA*, *KDR* were both significantly amplified in GBM samples compared with normal samples (p<0.001, Figure 3D, Supplementary Figure 1B, 1C, 1E). We also investigated the *KDR* methylation difference between GBM and normal samples with GSE50293 glioblastoma methylation data, results showed that there is no significant difference regarding to *KDR* methylation between GBM and normal samples (Supplementary Figure 1F). Results strongly suggested that increased *AURKA* mRNA level in glioblastoma is caused by *AURKA* copy number aberrant amplification. In summary, *AURKA* and *KDR* genes are hub driver genes in GBM oncogenesis process, *AURKA* and *KDR* encoding proteins are promised targets for GBM treatment.

### Virtual screening of small molecules inhibitors

**Figure 4:**
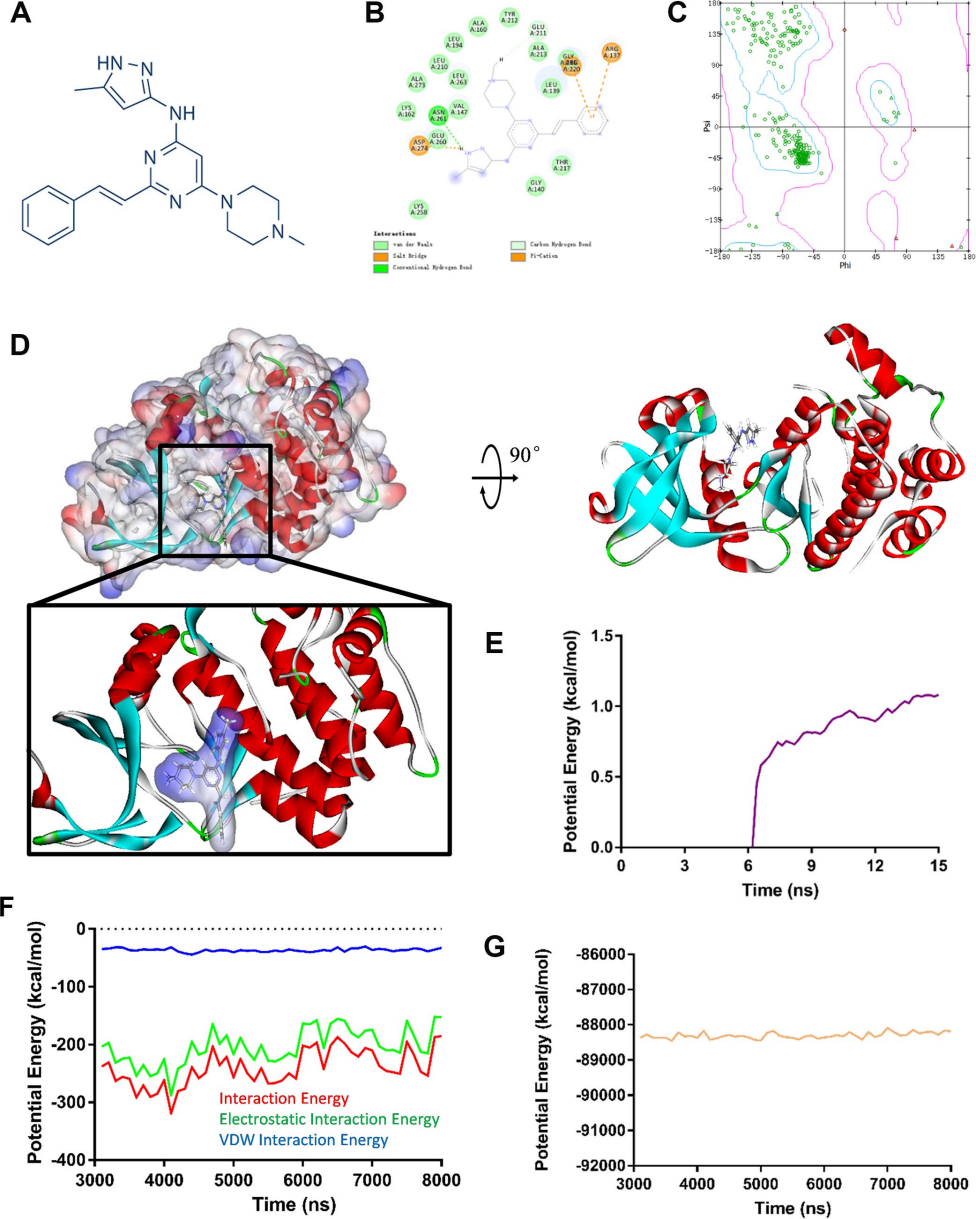
(**A**) ENMD-2076 chemical structure from virtual screening. (**B**) 2D inter-molecular interaction diagram of ENMD-2076 Aurora kinase A complex. (**C**) The Ramachandran diagrams of Aurora kinase A. (**D**) Schematic drawing of interactions between Aurora kinase A and ENMD-2076. (**E**) Average backbone root mean square deviations (RMSD) curve of ENMD-2076 Aurora kinase A complex. (**F**–**G**) Potential energy profiles of ENMD-2076 Aurora kinase A complex performed by molecular dynamic simulation.

Aurora kinase A is an enzyme encoded by the *AURKA* gene. A virtual screening was performed to identify most effective small molecules inhibitors targeting Aurora kinase A since *AURKA* was considered as the primary hub gene accounting for GBM oncogenesis. After calculated by LibDock, top 20 ranked molecules with LibDock scores were listed in Table 2. ZINC34885047 (ENMD-2076, chemical structure is shown in Figure 4A) is one of the top LibDock scores compounds, which demonstrate it has a gorgeous binding ability with Aurora kinase A.

**Table 2:** Top 20 ranked compounds predicted with LibDock.

**Number**	**Compounds**	**LibDock Score**	**Number**	**Compounds**	**LibDock Score**
1	ZINC03918087	138.344	11	ZINC03814434	118.793
2	ZINC58661275	134.926	12	ZINC43129461	118.487
3	ZINC06718723	134.376	13	ZINC03939511	118.159
4	ZINC34885047	131.973	14	ZINC72178031	117.923
5	ZINC15449300	131.33	15	ZINC06718665	117.787
6	ZINC34842284	130.825	16	ZINC38995988	115.722
7	ZINC13643145	128.306	17	ZINC16697102	114.91
8	ZINC28824607	126.122	18	ZINC35880991	114.177
9	ZINC14951647	124.408	19	ZINC00988000	113.465
10	ZINC28714095	119.2	20	ZINC58660958	111.534

### Evaluating ADMET (adsorption, distribution, metabolism, excretion, toxicity) properties

The parameters included brain/blood barrier (BBB), human intestinal absorption, aqueous solubility, hepatotoxicity, cytochrome P450 2D6 (CYP2D6) binding and plasma protein binding (PPB) properties were predicted by ADME module (Supplementary Table 3). Among various compounds, ZINC34885047 (ENMD-2076) was considered as an ideal, effective, non-toxic inhibitor for both Aurora kinase A and VEGFR-2. ENMD-2076 had the ability to pass through BBB (Score: 2), which is an especially important prerequisite for anti-glioblastoma drug. Also, ENMD-2076 had a good absorption level (Score: 0), an excellent aqueous solubility (water temperature: 25°C, Score: 2) and a perspective plasma protein binding (PPB) property (Score: 0). Additionally, ENMD-2076 was also considered as a hepatic safe (Score: 0), non-carcinogen (Female rat score: 0, Male rat score: 0.003), non-mutagen (Score: 0), non-developmental toxic medication based on hepatotoxicity evaluation, National Toxicology Program (NTP), Ames mutagenicity (AMES) evaluation and developmental toxicity potential (DTP) property prediction, respectively. Therefore, ENMD-2076 was predicted as a non-toxic and safe medication for further research.

### Ligand binding site analysis and molecular dynamics simulation

The molecular structure of ENMD-2076, Aurora kinase A complex was generated by CDOCKER module (Figure 3D), its CDOCKER interaction energy was -54.294 kcal/mol. Molecular docking results revealed that ENMD-2076 formed 2 pair of hydrogen bonds and 3 pairs Pi interactions with Aurora kinase A (Supplementary Table 4, Figure 3B), which demonstrated the complex is highly stable. Ligand binding site analysis was also carried out regarding to ENMD-2076, VEGFR-2 complex by the same protocol. The CDOCKER interaction energy of ENMD-2076, VEGFR-2 complex was -52.376 kcal/mol. The molecular structure of ENMD-2076, VEGFR-2 complex revealed that ENMD-2076 formed 2 pair of hydrogen bonds and 5 pairs Pi interactions with VEGFR-2 (Supplementary Figure 2B, 2C, Supplementary Table 4). The Ramachandran plot indicated that the crystal structure of Aurora kinase A and VEGFR-2 employed in this study was stable and obeyed theoretical predication (Figure 3C, Supplementary Figure 2A).

Molecular dynamics simulation was performed to evaluate ENMD-2076, Aurora kinase A complex and ENMD-2076, VEGFR-2 complex stability under dynamic conditions, respectively. The initial conformations were obtained from CDOCKER molecular docking experiment. The RMSD curves and potential energy of ENMD-2076, Aurora kinase A complex revealed that the complex trajectory reached equilibrium after 15 ns and complex potential energy got stabilized with the time (Figure 3E–3G). Similar results were also observed regarding to ENMD-2076, VEGFR-2 complex (Supplementary Figure 2D–2F). In conclusion, molecular dynamics simulation results indicated that ENMD-2076 interacts with Aurora kinase A and VEGFR-2 steadily.

### ENMD-2076 inhibited the proliferation of glioblastoma cell lines *in vitro*

**Figure 5:**
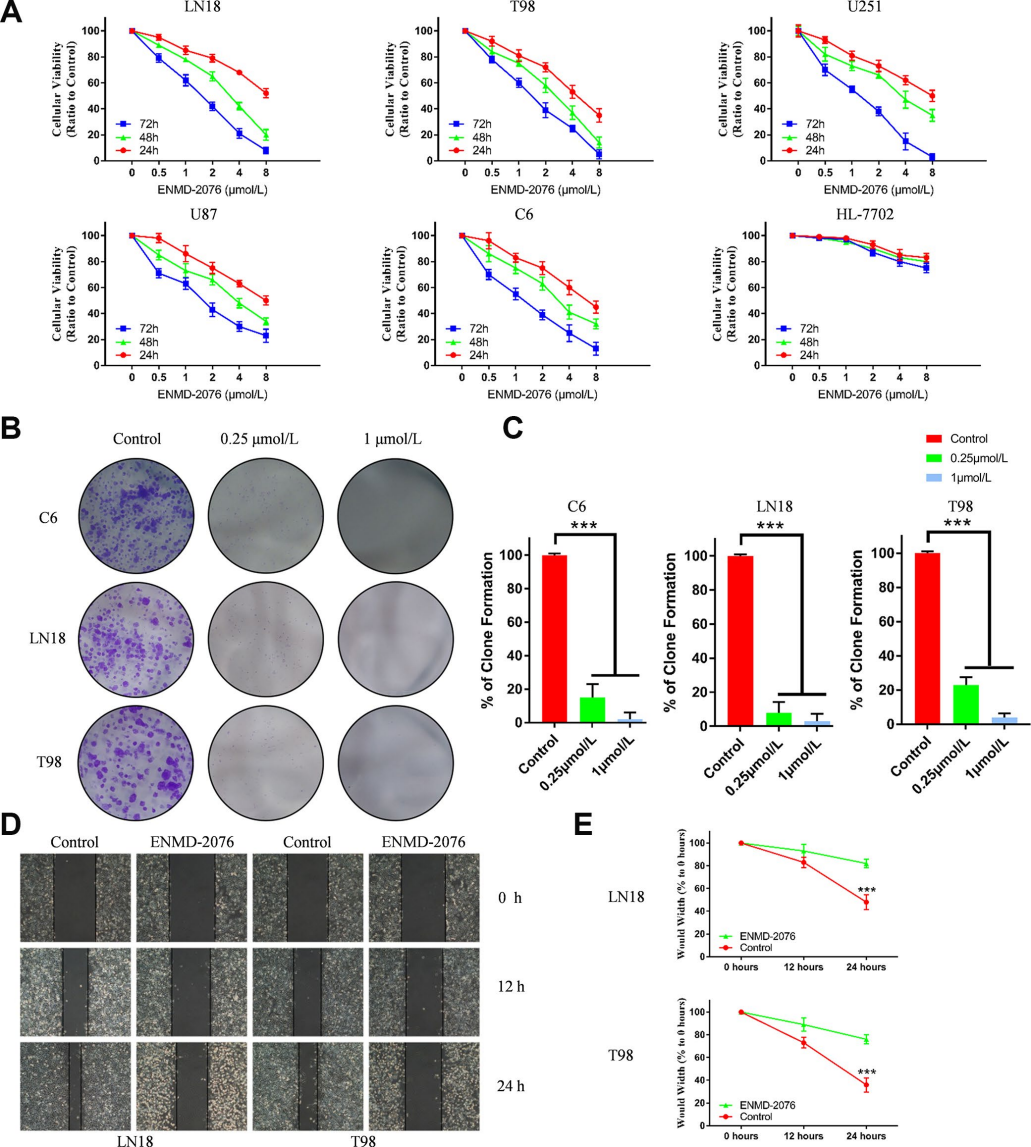
Data were presented as the mean ± SD. * p<0.05, *** p<0.01. (**A**) The results of cell proliferation in 5 glioblastoma cell lines and HL-7702 treated with different doses of ENMD-2076 (0, 0.5, 1, 2, 4, 8μM) for 24, 48, 72 hours. (**B**–**C**) Colony formation assay results of ENMD-2076 anti-proliferative effects in C6, LN18 and T98G cells for 2 weeks. (**D**–**E**) ENMD-2076 suppressing the migration of LN18, T98G cells in the scratch assay. The wound-healing images represented the migration capacity of glioblastoma cells.

MTT assay was used to evaluate the anti-proliferative effect of ENMD-2076. LN18, T98G, U87, U251, C6 cells were treated with different doses of ENMD-2076 (0, 0.5, 1, 2, 4, 8μmol/L) for 24, 48, 72 hours. As shown in Figure 5A, ENMD-2076 inhibited the proliferation of glioblastoma cells in a dose dependent manner and time dependent manner. Normal cell line (HL-7702) was also used to explore the ENMD-2076 toxicity in normal cells. The results of MTT assay revealed that ENMD-2076 (0.5, 1, 5μmol/L) did not inhibit the proliferation of HL-7702 in a dose dependent manner and time dependent manner. Therefore, ENMD-2076 possessed limited toxicity to normal cells.

Colony formation assay (CFA) was performed to evaluate the anti-proliferative effect of ENMD-2076 (0.25, 1μmol/L) in C6, LN18 and T98G cells. After 2 weeks, results showed that ENMD-2076 treated group significantly inhibited proliferation of glioblastoma cell lines in a dose-manner (Figure 5B, 5C). Thus, the results indicated that ENMD-2076 inhibited the proliferation of glioblastoma cell lines *in vitro.*

### ENMD-2076 suppressed the migration and invasion of glioblastoma cell lines *in vitro* by inhibiting EMT (epithelial-mesenchymal transition) process

**Figure 6:**
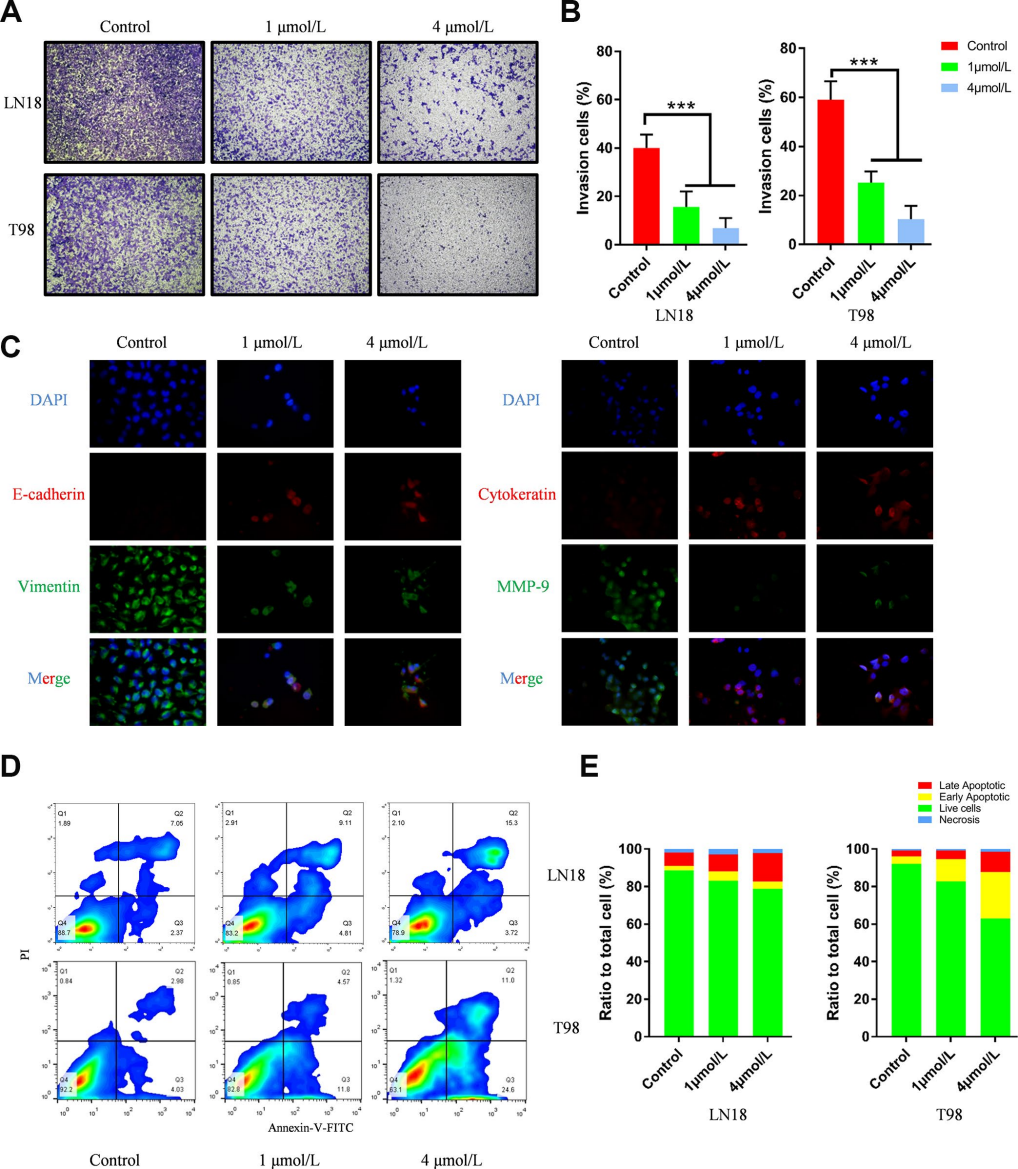
(**A**–**B**) ENMD-2076 suppressing the invasion ability of LN18, T98G cells in the Transwell assay. 5 random fields were counted for each condition. (**C**) Protein levels of E-cadherin, Vimentin, Cytokeratin and MMP-9 evaluated by immunofluorescence demonstrated ENMD-2076 inhibit glioblastoma invasion by suppressing EMT. (**D**–**F**) Flow cytometry results showed that ENMD-2076 induced glioblastoma cells (LN18, T98G) apoptosis. ENMD-2076 showed pro-apoptotic effects on LN18, T98G cells.

Scratch assay and Transwell assay were performed to evaluate the ability of ENMD-2076 in inhibiting the migration and invasion of glioblastoma cells. In the scratch assay, the wound-healing images represented the migration capacity of glioblastoma cells. Quantification line chart indicated the migration ability of glioblastoma cells. Results showed that ENMD-2076 significantly suppressed the migration of LN18, T98G cells (Figure 5D, 5E). As shown in Figure 6A, 6B, representative, results of Transwell assay showed that ENMD-2076 significantly suppressed the invasion of LN18, T98G cells. Additionally, the expression level of EMT (epithelial-mesenchymal transition) landmark proteins including E-cadherin, Vimentin, Cytokeratin and MMP-9 were also assessed by immunofluorescence, results showed that ENMD-2076 inhibit glioblastoma migration and invasion ability by suppressing EMT process.

### ENMD-2076 induced glioblastoma cells apoptosis, cell cycle G2-M phase arrest by inhibiting PI3K/AKT/mTOR pathway

**Figure 7:**
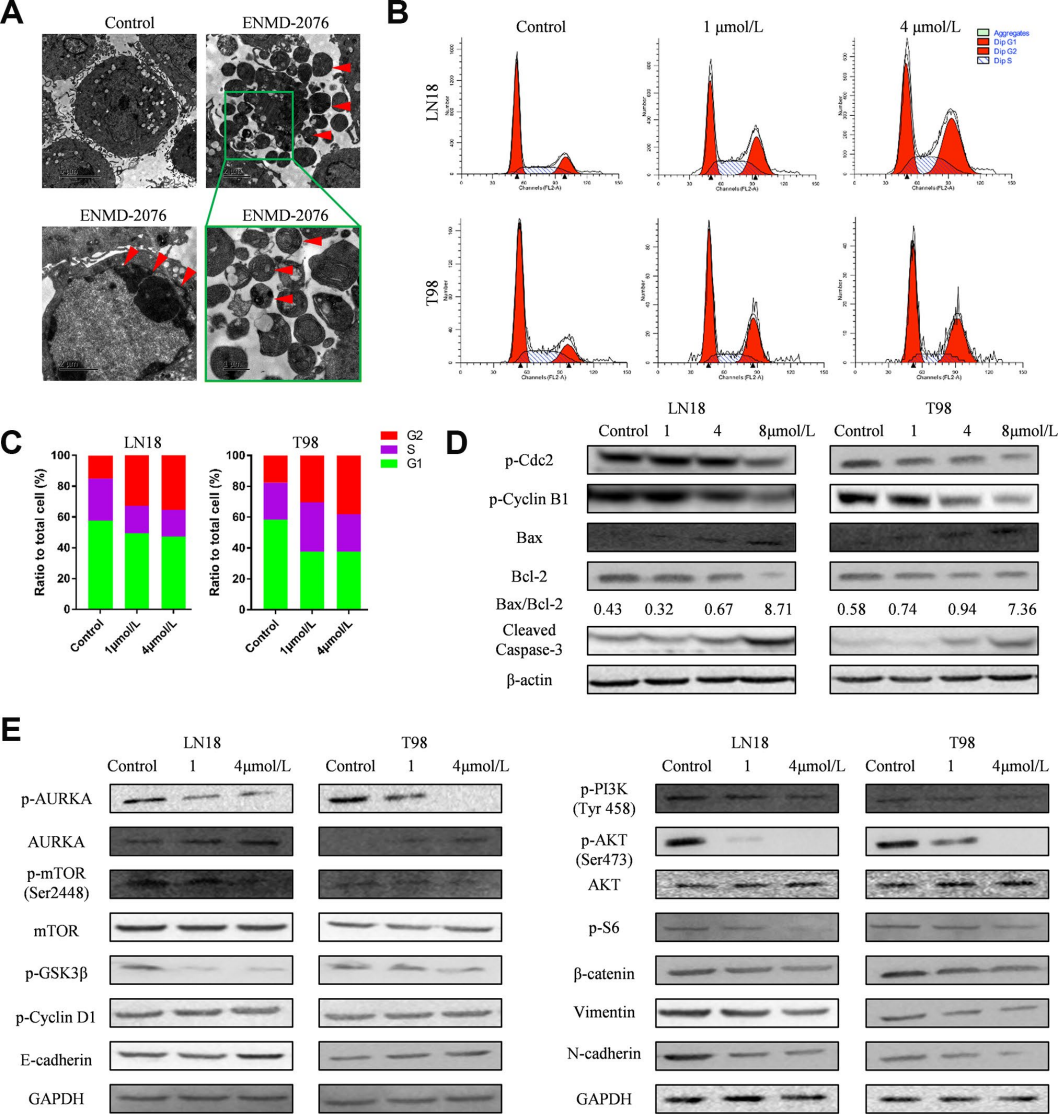
(**A**) Transmission Electron Microscope (TEM) images showed that ENMD-2076 induces apoptosis bodies and pyknosis formation in U87 cells. (**B**–**C**) Flow cytometry showed that ENMD-2076 induced cell cycle arrest in G2-M phase for LN18, T98G cells. (**D**) Western blot analysis showed that ENMD-2076 increased the expression of Caspase-3 and Bax in LN18, T98G cells and decreased the expression of anti-apoptotic Bcl-2. Western blot analysis also showed the effects of ENMD-2076 on the phosphorylation levels of Cdc2, cyclin B1 and cyclin D1 in LN18 and T98G cell lines. (**E**) Western blot analysis showed that ENMD-2076 suppressing PI3K/Akt/mTOR signaling pathway in LN18, T98G cells.

Flow cytometry was used to examine the apoptosis of glioblastoma cells. LN18, T98G cells were treated with various concentration of ENMD-2076 (1μmol/L and 4μmol/L) for 24h. Flow cytometry results showed that ENMD-2076 had pro-apoptotic effects on LN18, T98G cells (Figure 6D, 6E). Transmission Electron Microscope (TEM) images showed that ENMD-2076 induces apoptotic changes in U87 cells, including smooth cell surface, lost microvilli and Marginal condensation of heterochromatin in nucleus. Besides, treatment with ENMD-2076 induces apoptosis bodies formation in U87 cells, which was enveloped with membranes, encapsulating nuclear debris and some cytoplasmic components. Meanwhile, pyknosis was also observed in U87 cells treated with ENMD-2076 24h, which strongly suggested that cell cycle arrest has been induced by ENMD-2076 (Figure 7A). Additionally, the expression levels of pro-apoptotic proteins including Cleaved Caspase-3, Bax and anti-apoptotic containing Bcl-2 were evaluated by western blot analysis. Western blot analysis revealed that treating ENMD-2076 for 24h increased the expression of Cleaved Caspase-3 and Bax in LN18, T98G cells as well as decreased the expression of anti-apoptotic Bcl-2 and thereby inducing glioblastoma cells apoptosis, the ratio of Bax/Bcl-2 increased in a dose-manner (Figure 7D).

To further investigate the mechanism of ENMD-2076 anti-glioblastoma effect, we also carried a series of experiments to test the influence of ENMD-2076 upon cell cycle and signaling pathways of glioblastoma cells. LN18, T98G cells were incubated with ENMD-2076 for 24h. Flow cytometry analysis was performed to evaluate cell cycle arrest. Results indicated that ENMD-2076 induced cell cycle arrest in G2-M phase (Figure 7B, 7C). The percentage of G2-M cells in the ENMD-2076 4μmol/L group was significantly higher than control group. The endogenous cyclins expression levels were examined to validate flow cytometry analysis results. Western blot analysis revealed that ENMD-2076 reduced the expression levels of p-cyclin B1 and p-CDC2 in LN18, T98G cell line, which demonstrated that the transition of S to G2-M was obstructed. However, p-cyclin D1, which is a crucial protein for cell cycle G1/S transition, hasn’t been affected significantly. Our results indicated that ENMD-2076 induced the G2-M phase cell cycle arrest of glioblastoma cells by down-regulating cyclin B1 phosphorylation (Figure 7E, Supplementary Figure 3). Additionally, PI3K/Akt/mTOR signaling pathway was also fully investigated in this study, results showed that ENMD-2076 inhibits p-AURKA, p-PI3K, p-Akt, p-mTOR level and thereby suppressing PI3K/ Akt/mTOR signaling pathway to block EMT process. However, we didn’t observe a significant change regarding to AKT and mTOR expression level. Besides, ENMD-2076 induces glioblastoma cells apoptosis via inhibiting Akt/GSK3β/β-catenin signaling pathway. The proposed molecular mechanism of this study was displayed in Figure 9.

### ENMD-2076 showed perspective therapeutic effect *in vivo*

**Figure 8:**
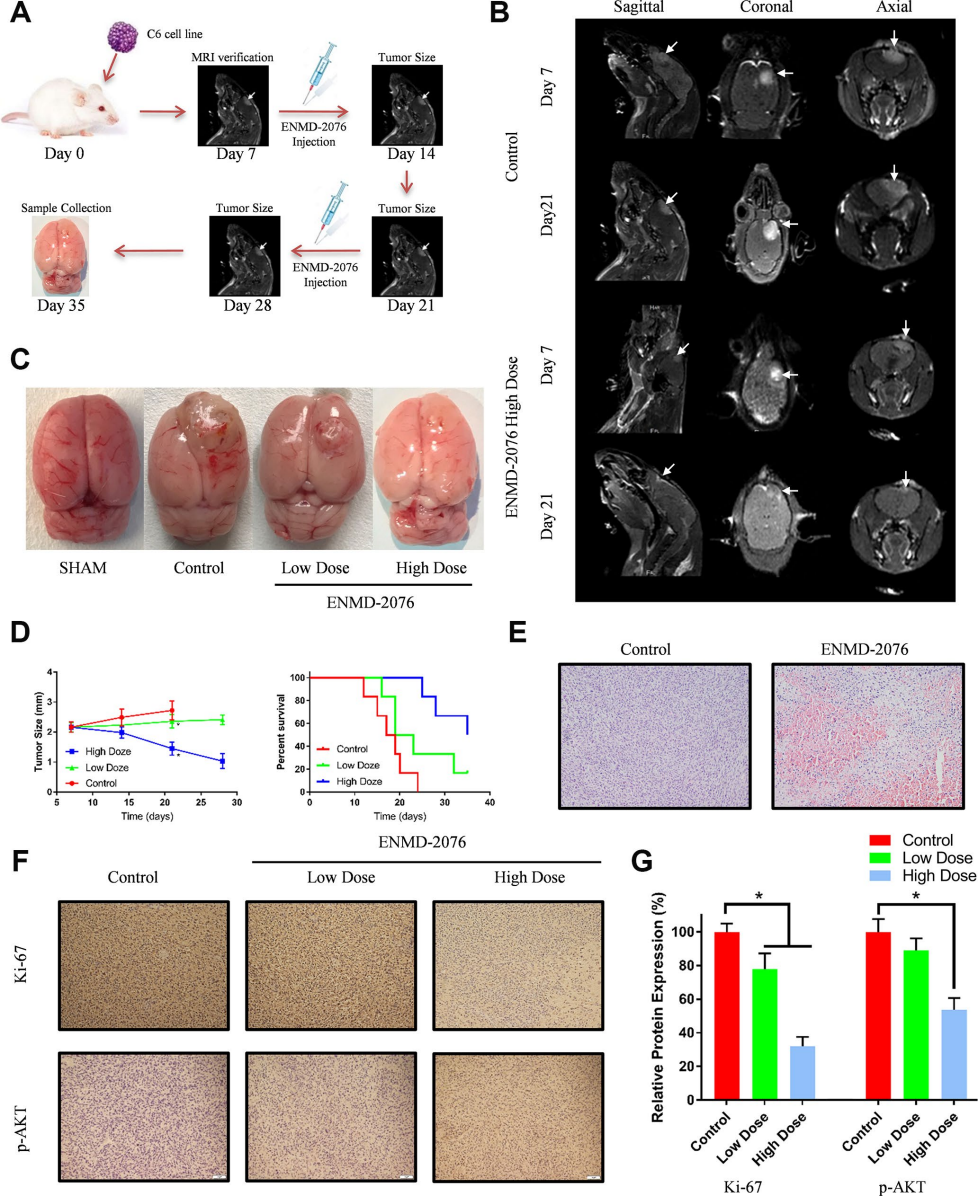
(**A**) The overall framework of animal experiment. C6 cells were intracranial injected on day 0. Magnetic resonance imaging (MRI) was used to evaluate the tumor growth on day 7. (**B**) MRI examination with different planes (sagittal, coronal, axial). (**C**) The representative samples showing the tumor volumes in different groups. (**D**) Tumor size and percent survival curves in different groups. (**E**) Histologic features of control group and ENMD-2076 treated group. (**F**–**G**) Immunohistochemistry analysis of ki-67, p-AKT in the different groups.

With an intracranial tumor model, we evaluated whether ENMD-2076 had therapeutic anti-glioblastoma effects *in vivo*. 7 days after C6 cells intracranial injection, magnetic resonance imaging (MRI) was used to evaluate the tumor growth (Figure 8A, 8B). After MRI verification, the 24 tumor-bearing rats were randomly subdivided into 3 groups: control group (8 rats), low dose group (50 mg/kg, 8 rats) and high dose group (200 mg/kg, 8 rats). Rats received ENMD-2076 treatment for 28 days (4 weeks) after tumor implantation. As shown in Figure 8C, 8D, ENMD-2076 decreased growth rate of tumor volume in a dose manner (P<0.01). Kaplan-Meier survival analysis also indicated that ENMD-2076 significantly prolonged the survival time of tumor-bearing rats as well as inhibiting tumor growth (p<0.01). Histological results suggested that glioblastoma tissue in ENMD-2076 treatment group form more necrosis and inflammation regions compared with control group, shown in Figure 8E. Immunohistochemical analysis showed ENMD-2076 decreased the expression of Ki-67, p-AKT, p-S6 in a dose manner (Figure 8F, 8G, Supplementary Figure 4A). Additionally, no pathological changes have been observed in the brains, lungs, livers, kidneys and hearts of the rats treated with high doze ENMD-2076, which also indicate ENMD-2076 is a safe medication for clinical application (Supplementary Figure 4B).

**Figure 9:**
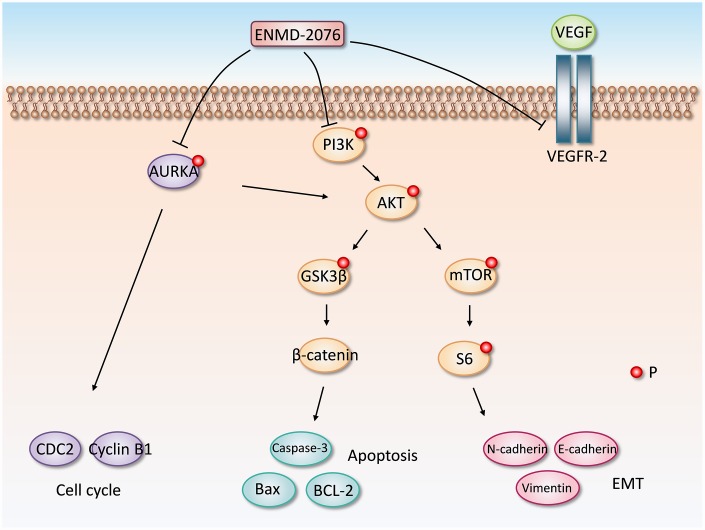
**The proposed molecular mechanism of ENMD-2076 anti-glioblastoma effect.**

## DISCUSSION

Glioblastoma is the most common type of malignant brain tumor with poor prognosis and high mortality. More recently, bioinformatics technology and structure biology were effectively and systematically used to identify specific targets in malignant tumors [4, 16]. WGCNA analysis is a kind of data mining methods developed in recent years characterized by screening out significant modules and gene signatures associated with clinical traits and phenotypes [9, 17]. Gene expression profiles from the GSE50161 were extracted to perform bioinformatics analysis in this study. With WGCNA analysis, we successfully identified that the significant gene cluster (blue module) was highly correlated with glioblastoma. Module-trait relationship showed that the relationship between blue module and GBM was higher than other module-trait relationships. Furthermore, PPI network analysis demonstrated that *AURKA* and *KDR* genes were hub driver genes of glioblastoma. Meanwhile, the functional and pathway enrichment analysis of blue module was highly concordant with the findings that cell cycle pathway and cell proliferation ontology functions were regulated abnormally in GBM. After combining the results of bioinformatics analyses, we selected *AURKA* and *KDR* as targets for further investigation.

Aurora A kinase is a member of serine-threonine kinases family, which is encoded by *AURKA*. It takes part in the process of cell proliferation by regulating mitosis and chromosomal segregation [18]. *AURKA* is constituted with a protein kinase domain, a N-terminal domain and C-terminal domain. The activity of *AURKA* peaks in the transition of G2 phase to M phase at cell cycle progresses. Abnormal amplification or overexpression of *AURKA* could be associated with high occurrence of cancers including gastric cancer, ovarian cancer, breast cancer, etc [19–22]. Researchers have discovered that *AURKA* is involved in tumorigenesis with multiple mechanisms. RAS-association domain family 1, isoform A (*RASSF1A*) is a tumor suppressor gene which accounts for M-phase cell cycle arrest and stabilizing microtubules [23]. *AURKA* leads to uncontrolled proliferation of cancer cells by phosphorylating RASSF1A and disrupting RASSF1A interactions with microtubules. In addition, *AURKA* overexpression could abolish M-phase cell cycle arrest by inhibiting the ability of RASSF1A [24]. Furthermore, *AURKA* could inhibit the apoptosis of cancer cells by decreasing pro-apoptotic modulators (Caspase-3, Bax) and increasing anti-apoptotic regulators (Bcl-2). *AURKA* promotes epithelial-mesenchymal transition by inhibiting the expression of E-cadherin and β-catenin [25–27]. *AURKA* overexpression could also increase the expression of MMP-2, MMP-7, MMP-9, leading to tumor metastasis by degrading extracellular matrix proteins [28, 29]. So, *AURKA* is a promising target in dealing with cancer.

*KDR* is a VEGF receptor known as vascular endothelial growth factor receptor 2 (VEGFR-2) [30, 31]. It is involved in angiogenesis and vasculogenesis. Aberrant angiogenesis is critical in the process of tumor growth and metastasis [32]. VEGFR-2 could enhance vessel permeability and stimulate endothelial cell proliferation, differentiation and migration [33–35]. Excessive VEGFR-2 activation is an important driver of tumor angiogenesis and VEGFR-2 is significantly increased on the tumor vasculature. So, it has emerged a potential approach to anticancer therapy by blocking VEGF/ VEGFR-2 signaling pathway [36]. Many small molecular drugs blocking VEGFR-2 such as ramucirumab and aflibercept have been demonstrated as prospective anticancer drugs.

In this study, we discovered that *AURKA* and *KDR* mRNA were significantly overexpressed in GBM samples. DNA copy number expression of *AURKA* and *KDR* were higher in GBM samples, either. However, there is no significant difference of *KDR* methylation between GBM and normal samples and *AURKA* doesn’t possess methylation site. The results indicated that the reasons of excessive *AURKA* and *KDR* expression might be aberrant DNA amplification rather than methylation. We also employed survival curve analysis to estimate the relationship between GBM prognosis and *AURKA*, *KDR* expression. The results indicated that GBM patients with high expressed *AURKA* and *KDR* had poor prognosis.

After identifying *AURKA* and *KDR* as potential therapeutic targets, we successfully found the small molecular compound, ENMD-2076, could effectively inhibit *AURKA* and VEGFR-2 activities through virtual screening technique. Molecular docking study indicated that ENMD-2076 could bind to *AURKA* and VEGFR-2 steadily. In addition, our results showed that ENMD-2076 has medium level to pass brain/blood barrier and has a good absorption level. Moreover, ENMD-2076 was not predicted with toxic properties including hepatotoxicity, rodent carcinogenicity, mutagenicity and developmental toxicity potential.

ENMD-2076 is a kind of novel oral bioavailable small-molecules which is an active aurora kinase and tyrosine kinase inhibitor [37, 38]. The main targets of ENMD-2076 include Aurora A kinase, Aurora B kinase, VEGFR-2 and VEGFR-3. Previous *in vitro* studies have demonstrated that ENMD-2076 showed antitumor activities against several human cancer cell lines including breast cancer, melanoma, colorectal cancer, etc [15, 39, 40]. ENMD-2076 inhibited the proliferation of breast cancer and colorectal cancer lines. It also induced apoptosis and G2 cell-cycle arrest of breast cancer and colorectal cancer lines [15, 40]. ENMD-2076 had better cytotoxic and apoptotic effects if the cancer cell lines possessed increased p53 expression and p53 mutation. Previous *in vivo* experiments demonstrated ENMD-2076 induces regression or complete inhibition in tumor xenograft models including colorectal cancer, breast cancer and multiple myeloma [39]. Several Phase I and II clinical trials involving ENMD-2076 showed that it is a promising antitumor drug in dealing with ovarian cancer, multiple myeloma, etc [41–43]. However, the antitumor activity of ENMD-2076 against glioblastoma has not been evaluated. Thus, we examine its antitumor activities against glioblastoma in this study.

In this study, we demonstrated that ENMD-2076 could inhibit the proliferation of GBM cell lines. ENMD-2076 suppressed the migration and invasion of GBM cells by blocking EMT process. In addition, we investigated ENMD-2076 induced cell cycle arrest of glioblastoma cells in G2-M phase. ENMD-2076 could also induce apoptosis of glioblastoma cells by decreasing anti-apoptotic protein Bcl-2 and increasing pro-apoptosis proteins Cleaved Caspase-3, Bax. Our *in vivo* experiments discovered that ENMD-2076 increased the median survival time of tumor-bearing rats and decreased growth rate of tumor volume in a dose manner. Mechanism study in our experiment showed that ENMD-2076 down-regulated the activation of PI3K/AKT/mTOR signaling pathways. It is well-known that abnormal activation of PI3K/AKT/mTOR signaling pathways are responsible for the proliferation and metastasis of GBM cells. Abnormal activation of this signal transduction pathway also leads to reduced apoptosis of GBM cells. As a result, ENMD-2076 could inhibit GBM cells proliferation, migration and induce apoptosis via suppressing PI3K/AKT/mTOR signaling pathways.

## CONCLUSIONS

In conclusion, 12 gene modules were identified from the dataset GSE50161 with WGCNA analysis. *AURKA* and *KDR* in the blue module were recognized as key therapeutic targets for GBM. ENMD-2076 was found as a potent inhibitor for Aurora A kinase and VEGFR-2 through virtual screening technique. *In vitro* and *in vivo* studies demonstrated that ENMD-2076 is a promising and safe drug in dealing with glioblastoma.

## MATERIALS AND METHODS

### Microarray data

Microarray datasets were downloaded from National Center for Biotechnology Information Gene Expression Omnibus (GEO) (http://www.ncbi.nlm.nih.gov/geo). The series accession number of gene expression profile dataset was GSE50161. The profile contains 13 normal brain samples and 34 glioblastoma samples. Log2 transformation was performed to obtain the standardized values of expression.

### WGCNA analysis

WGCNA analysis was used to identify corresponding expression modules. In R platform, hierarchical cluster analysis was firstly conducted to check the heterogeneity of samples. The network construction soft threshold power was then defined to get access to real biological network state. Next, pickSoftThreshold function of WGCNA package was implemented and performed to calculate soft threshold power of the gene expression profile. The soft threshold power was selected when the mean connectivity was almost 0 and the scale-free topology fit index value was almost 0.9. Then, weighted gene co-expression networks were constructed and the gene modules were identified. Dynamic tree cutting algorithm was used to define different gene modules. Eigengenes adjacency was calculated to evaluate the interaction of various gene modules. Heat map was also employed to visualize the interaction among different gene modules. After obtaining different gene modules by WGCNA, module-trait relationships were estimated to identify the correlation between modules and glioblastoma. Pearson’s correlation coefficient was also calculated to evaluate correlation between module eigengene and phenotype. After identifying key gene module, further analysis regarding to the identified module was conducted. Relationship between Gene Significance (GS) and Module Membership (MM) was evaluated. A scatter diagram was plotted to display the correlation between glioblastoma and selected gene module.

### GO (Gene ontology), KEGG (Kyoto Encyclopedia of Genes and Genomes) pathway and GSEA analysis (Gene Set Enrichment Analysis)

Functional annotation and interpretation were performed to discover biological meaning of genes. We used The DAVID database (Database for Visualization, Annotation and Integrated Discovery, http://david.abcc.ncifcrf.gov/) to get comprehensive functional annotation. The cutoff criteria was considered as *p < 0.05*. GO analysis is a powerful tool to identify enriched biological themes including molecular function and biological process. Kyoto Encyclopedia of Genes and Genomes (KEGG) is a useful method to analyze information about pathway relationships. In this study, we performed GO analysis and KEGG pathway in DAVID database to find out statistical significant data. Additionally, GSEA analysis was also performed to find functional annotation and interpretation. Based on the size of the set, a normal enrichment score (NES) is evaluated for each gene set, FDR and nominal p value were calculated as the cut-off criteria.

### Protein-protein interaction (PPI) network

PPI analysis was conducted on an online database Search Tool for the Retrieval of Interacting Genes (STRING; http://string-db.org/). In addition, we used Cytoscape software to identify densely connected regions with Molecular Complex Detection (MCODE). MCODE recognize most significant module by number of nodes > 9 and MCODE scores > 8. The significant difference was considered as P < 0.05. PPI network analysis results were also displayed as Circos plot, which was performed by Circos package in R platform.

### Clinical patients’ datasets

We downloaded the clinical glioblastoma patients data from The Cancer Genome Atlas (TCGA, https://cancergenome.nih.gov/) and Chinese Glioma Genome Atlas (CGGA, http://www.cgga.org.cn/). 137 TCGA patient (females: 51, males: 86), 325 CGGA patients (females: 122, males: 203) were included and their gene expression data were obtained. Patients were divided into 2 groups, respectively: low-expressed group and high-expressed group. Clinical parameters such as overall survival (OS) and progression- free survival (PFS) were considered as prognostic outcome of glioblastoma patients to determine if *AURKA* and *KDR* expression have a prognostic impact upon GBM patients.

### Molecular docking and absorption, distribution, metabolism, excretion, toxicity prediction

LibDock and CDOCKER modules of Discovery Studio were performed in the molecular docking study. LibDock is a high-throughput drug virtual screening algorithm, which can identify potential ligands for a specific receptor from compounds library. In the docking process, the ligands were able to flex while the receptor was held rigid. The CDOCKER could calculate energy to indicate ligand binding affinity for each complex pose. We got the crystal structure of *AURKA* (*AURKA* ID: 5DT0, 2.05 Å) in the Protein Data Bank. Then, we generally got rid of crystal water molecules and added hydrogen atoms to reduce the negative effects of fixed water molecules on the receptor-ligand complex formation. The ligands were permitted to attach to residues within binding site spheres during the docking process. On the basis of CDOCKER interaction energy, each test molecule in different poses were analyzed and generated. Based on the lowest energy representative of each cluster, the cluster ranking was performed. Interactions between ligands and protein were analyzed and visualized in DS4.5 (Discovery Studio 4.5). ADME (absorption, distribution, metabolism, excretion) module of Discovery Studio 4.5 was employed to calculate the absorption, distribution, metabolism, excretion of selected compounds. TOPKAT (Toxicity Prediction by Komputer Assisted Technology) modules of DS4.5 was also conducted to calculate the toxicity and other properties of all selected compounds, including their aqueous solubility, blood-brain barrier (BBB) penetration, cytochrome P450 2D6 (CYP2D6) inhibition, hepatotoxicity, human intestinal absorption, plasma protein binding (PPB) level, rodent carcinogenicity, Ames mutagenicity and developmental toxicity potential. These pharmacological properties were fully considered when selecting drug candidates for Aurora A kinase and VEGFR-2.

### Molecular dynamics simulation

The best binding conformations of each Compounds-Aurora A kinase complex were selected and prepared for molecular dynamics simulation. The ligand-receptor complex was put into an orthorhombic box and solvated with an explicit periodic boundary solvation water model. In order to simulate the physiological environment, solidum chloride were added to the system with the ionic strength of 0.145. Then, the system was subjected to the CHARMm forcefield and relaxed by energy minimization (500 steps of steepest descent and 500 steps of conjugated gradient), with the final RMS gradient of 0.289. The system was slowly driven from an initial temperature of 50 K to the target temperature of 300 K for 2 ps and equilibration simulations were run for 5 ps. Molecular dynamics simulations (production) were performed for 25 ps with time step of 1 fs. The simulation was performed with the NPT (normal pressure and temperature) system at a constant tem-perature of 300 K. The particle mesh ewald (PME) algorithm was used to calculate long range electrostatics, and the linear constraint solver (LINCS) algorithm was adapted to fix all bonds involving hydrogen. With initial complex setting as a reference, the trajectory was determined for structural properties, root mean-square deviation (RMSD), and potential energy by using trajectory protocol in Discovery Studio 4.5 (San Diego, CA, USA).

### Reagents and antibodies

Anti- E-cadherin, Vimentin, Cytokeratin, MMP-9, Caspase-3, Bax, Bcl-2, p-Cdc2, p-Cyclin B1, p-Cyclin D1, p-PI3K, p-AKT, AKT, p-mTOR, mTOR, p-S6, p-AURKA, AURKA, p-GSK3β, β-catenin, N-cadherin and Ki-67 antibodies were purchased from Abcam (Cambridge, MA, USA). Other reagents were purchased from Sigma (St. Louis, MO, USA).

### Cell lines

Glioblastoma cell lines (U251, U87, C6, LN18, T98), human normal glial cells (HEB), human liver cells (HL-7702) were obtained from the American Type Culture Collection. We cultured these cell lines in the Dulbecco’s modified Eagle’s medium (DMEM, GE Healthcare Life Sciences, Logan, Utah, USA). 10% fetal bovine serum (FBS, Gibco, Thermo Fisher Scientific, Waltham, Massachusetts, USA) was added to the cell culture media as supplement. 5% CO_2_ and 37°C with humidified air were the parameters of incubator for cell cultivation. The C6 cell line was cultured in F-12K medium (Gibco) supplemented with 12.5% horse serum (HyClone), 2.5% FBS (HyClone), and penicillin-streptomycin (100 U/ml, HyClone) in a humidified atmosphere of 5% CO_2_ at 37 °C.

### Cell proliferation assay

The standard MTT assay was used to measure the proliferation of cells. Cells were seeded with a density of 500 cells/well in 96-well plates. Different doses of ENMD-2076 were added into each well for incubation with 24, 48, or 72 hours. We used phosphate-buffered saline (PBS) to dissolve the MTT reagent (Sigma, St. Louis, Missouri, USA). On the measurement day, the fresh medium was made. The fresh medium contained DMEM, 10% FBS and diluted MTT reagent (1:10, 10% MTT). The previous medium was replaced with fresh medium and the plates were incubated for 4 hours. The absorbance was measured at 570 nm using the ELx800 absorbance microplate reader (BioTek Instruments, Winooski, Vermont, USA).

### Western blotting

Lysis buffer was used to prepare cellular extracts. BCA protein assay kit was used to detect the total protein in each sample. 8-15% Sodium dodecyl sulfate-polyacrylamide gel electrophoresis (SDS-PAGE) was used to separate proteins in samples. Then, the proteins were transferred to polyvinylidene difluoride (PVDF) membranes. 10% nonfat dry milk in TBST buffer was used to block the membranes with 1 h. After blocking, the membranes were incubated with primary antibodies at 4°C overnight. On the second day, the membranes were washed with TBST 3 times. After washing, horseradish peroxidase conjugated secondary antibody was added to incubate the membranes for 2 h at room temperature. The enhanced chemiluminescence reagents were used to detect the protein signals. Syngene Bio Imaging tools were used to measure the belt densities.

### Apoptosis assay

6-well plates were used in the cell apoptosis assay. 2×10^5^ cells were seeded in every well. Various concentrations of ENMD-2076 were added to the 6-well plates for 24 h. Subsequently, Accutase detachment solution (Sigma, St. Louis, Missouri, USA) was used to harvest cells based on the manufacturer’s instructions. Annexin V-FITC and propidium iodide (PI) were used to stain cells. At the end of staining process, flow cytometry technique was used to analyze stained cells.

### Cell cycle measurement

6-well plates were used in the cell cycle measurement. The cells in the log growth phase were analyzed. 2×10^5^ cells were seeded in every well. Various concentrations of ENMD-2076 were added to the 6-well plates. After incubation with ENMD-2076, cells were resuspended by PBS and incubated with pre-cooled ethanol (70%) overnight. On the second day, 100 μg/mL ribonuclease was added and incubated for 0.5 h. Subsequently, PI working solution was used to stain cells for 0.5 h in the dark. Flow cytometry technique was used to analyze stained cells.

### Colony formation assay (CFA)

Cells were seeded in the cell culture plates. DMEM and 10% FBS were used to culture cells for 2 weeks. Different concentrations of ENMD-2076 were added to the cell culture plates. After 2 weeks of cultivation, methanol was used to fix the cells. Then, 0.5% crystal violet solution was used to stain the developed colonies. Microscopic examination was used to count colony with more than 50 cells.

### *In vitro* scratch assay

6-well cell culture plates were used in the scratch assay. Cells were cultured in the plates. When the degree of fusion reached 90%, a 1-mL pipette tip was introduced to scratch a consistent cell-free area manually. Then, DMEM was used to rinse the cell debris. Subsequently, the cell culture plates were treated with or without different concentrations of ENMD-2076. After 0, 12, 24 h, the images of the scratched region were taken. The scratch width and wounded area were calculated.

### Cell migration assay (Transwell)

Transwell chambers (Corning, New York, NY) were used to evaluate cell migration assay according to the manufacturer’s protocol. Different doses of ENMD-2076 were added. After 24 h incubation, the upper surfaces cells were wiped by cotton swabs of Transwell chambers. Methanol was used to fix the migrated cells which were located on the lower surfaces. At last, the invasive cells were counted in at least 5 randomly fields.

### Cell immunofluorescence

Cells were seeded in 6-well culture plates with sterilized coverslips. Different doses of ENMD-2076 were added to incubate with cells. After incubation, 4% paraformaldehyde was used to fix cells. 0.25% Triton X-100 was used to make cells permeable. Then, cells were incubated with primarily antibody at 4°C overnight in a humidified chamber. On the secondary day, secondary antibody combined with fluorescein was used. DAPI was used to stain the nuclei. At last, the cells were photographed under fluorescence microscope and fluorescent signals were evaluated.

### Experimental animals

Male/Female Wistar rats were purchased from the Animal Center of the Chinese Academy of Medical Science (Beijing, China). Animals were maintained under specific pathogen free conditions in standard housing cages. All animal experiments were approved by the Institutional Animal Care and Use Committee at the First Hospital of Jilin University and in compliance with relevant international codes. All the experiments were conducted in accordance with the guidelines issued by the committee.

### C6-glioma rats model

C6 cells were used for implantation in glioma rats model. The rats were anesthetized by intraperitoneal (*i.p*) injection of 10% chloral hydrate. After shaving, the rats were fixed on the stereotactic head holder. The surgical zone was disinfected with 0.1% povidone-iodine. 1×10^6^ C6 glioma cells in 10 μL of PBS were implanted into the right frontal lobe by stereotactic surgery. The coordinates were as follows: 3 mm lateral, 1 mm behind coronal suture, 4 mm depth. The infusion rate of C6 cells was 1 μL/min. Bone waxes were used to fill the burr holes after implantation. After surgery, rats were conventionally bred. With MRI verification on 7 days later after implantation, tumor-bearing animals were randomly divided into positive control group, low dose group (50 mg/kg) and high dose group (200 mg/kg).

### MRI scanning

MRI scans were used to evaluate tumor size on 7, 14, 21 and 28 days after implantation. 10% chloral hydrate (*i.p*) was used to anesthetize the rats. After tail intravenous injection with gadolinium contrast agents, sagittal sequence, coronal sequence and axial sequence were used to acquire T1 weighted MR images and calculate tumor size. Image J software was used to analyzed the volume of tumor.

### Histopathology and immunohistochemistry

On day 35 after implantation, the animals were fully anaesthetized and sacrificed. For histological examination, samples were fixed in 4% paraformaldehyde. The samples were processed for paraffin embedding. Tissue sections were stained with haematoxylin and eosin (H&E). For immunohistochemistry, tissues were blocked by bovine serum albumin (BSA) at room temperature. Then, tissues were incubated with primarily antibody (Ki-67, p-AKT, p-S6) at 4°C overnight. On the secondary day, secondary antibody was added at 37 °C for 30 min. At last, slides were photographed with a light microscope.

### Statistical analysis

SPSS 18.0 (SPSS Inc., Chicago, Illinois, USA) was used to analyze all statistic data. To analyze the quantitative data, independent-samples *t*-test or one-way analysis of variance (ANOVA) was performed. Significance was set as P value <0.05.

## Supplementary Material

Supplementary Figures

Supplementary Tables
