# Supplementary material for: Selected by gene co-expression network and molecular docking analyses, ENMD-2076 is highly effective in glioblastoma-bearing rats
Source: Aging (Albany NY). 2019 Nov 9;11(21):9738–66. doi: 10.18632/aging.102422 (PMC6874459; doi:10.18632/aging.102422)
Supplement: Supplementary Figures [file aging-11-102422-s002.pdf]

SUPPLEMENTARY FIGURES

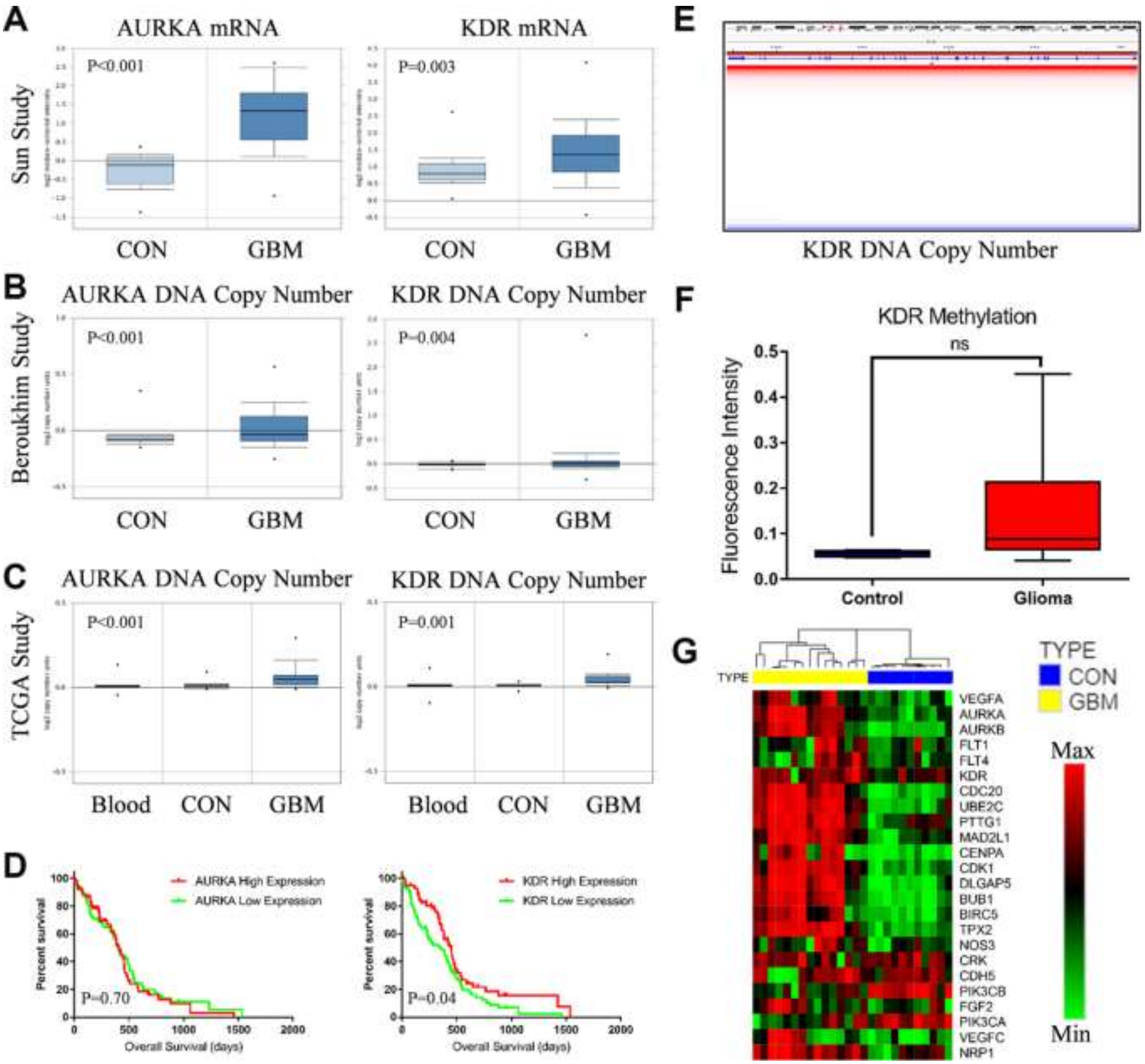

Supplementary Figure 1. (**A**) Based on Sun study, the mRNA expression of AURKA (p<0.001) and KDR (p=0.003) were investigated. (**B**) Based on the Beroukhim study, the DNA copy number of AURKA (p<0.001) and KDR (p=0.004) were investigated. (**C**) Based on the TCGA study, the DNA copy number of AURKA (p<0.001) and KDR (p=0.001) in blood, control and GBM were investigated. (**D**) TCGA dataset, the OS difference between high-expression and low expression AURKA. (**E**) The DNA copy number of KDR. (**F**) The methylation level of KDR between control and glioma. ns: non-significance. (**G**) The heat map of gene expression levels between GBM and normal samples. The right side of heat map indicated the colors of correlation (green represented negatively correlated, red represented positively correlated).

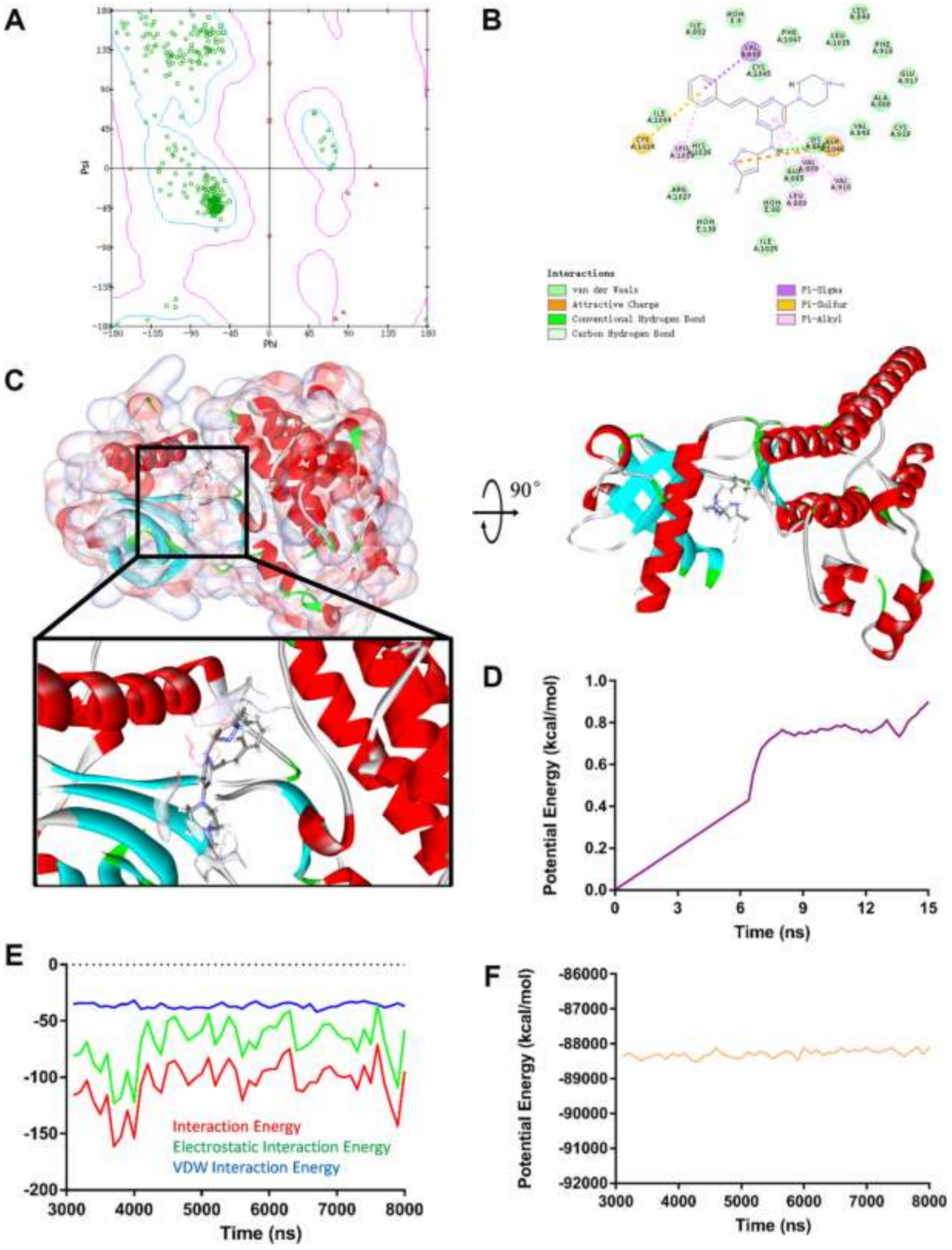

Supplementary Figure 2. (**A**) The Ramachandran diagrams of VEGFR-2 (encoded by *KDR*). (**B**) 2D inter-molecular interaction diagram of ENMD-2076, VEGFR-2 complex. (**C**) Schematic drawing of interactions between ENMD-2076 and VEGFR-2. (**D**) Average backbone root mean square deviations (RMSD) curve of ENMD-2076, VEGFR-2 complex. (**E–F**) Potential energy profiles of ENMD-2076 VEGFR-2 complex performed by molecular dynamic simulation.

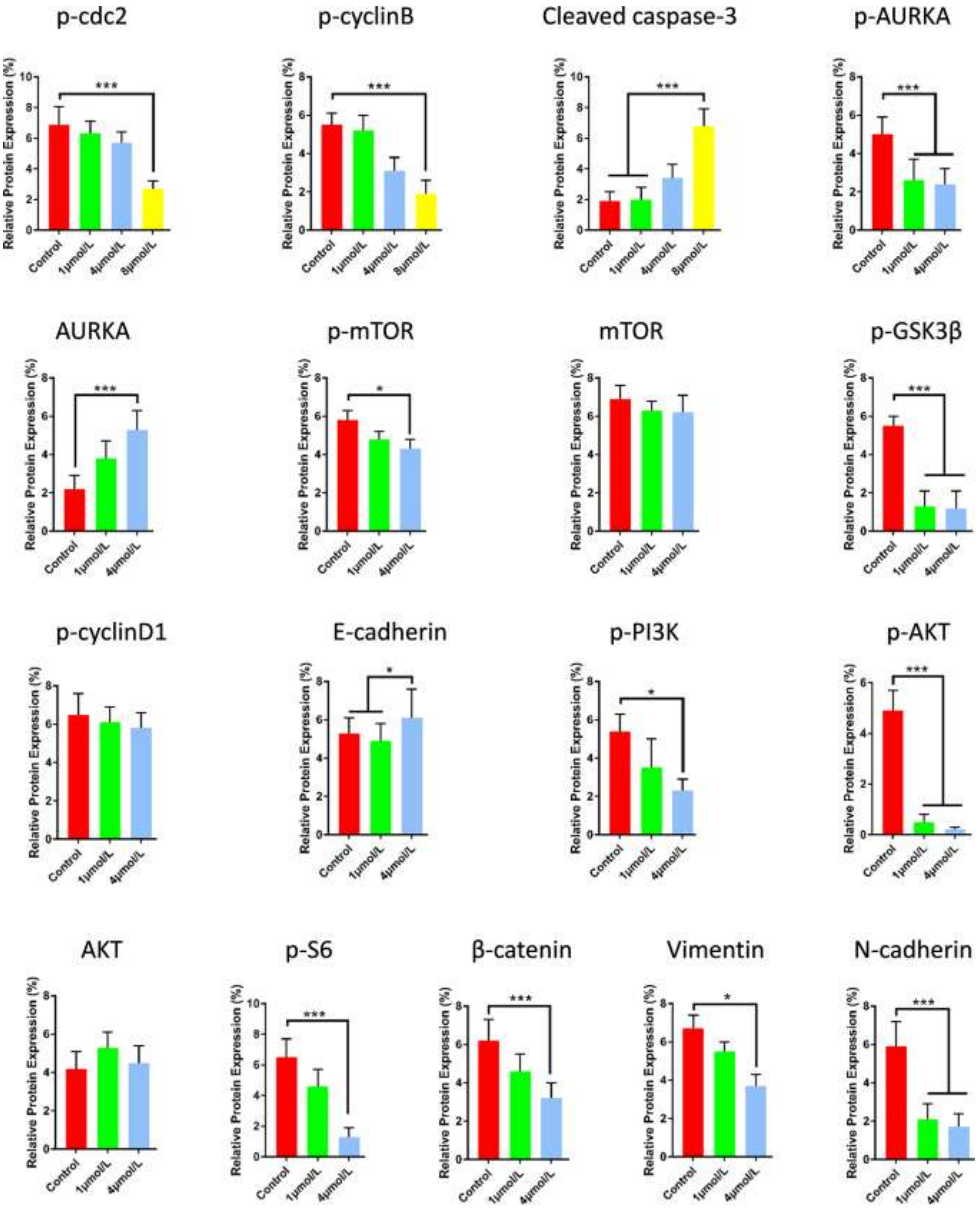

Supplementary Figure 3. Western blot showed that the effect of ENMD-2076 on the expression of the p-cdc2, p-cyclinB, Cleaved caspase-3, P-AURKA, AURKA, p-mTOR, mTOR, p-GSK3β, p-cyclinD1, E-cadherin, p-P13K, p-AKT, AKT, p-S6, β-catenin, Vimentin and N-cadherin.

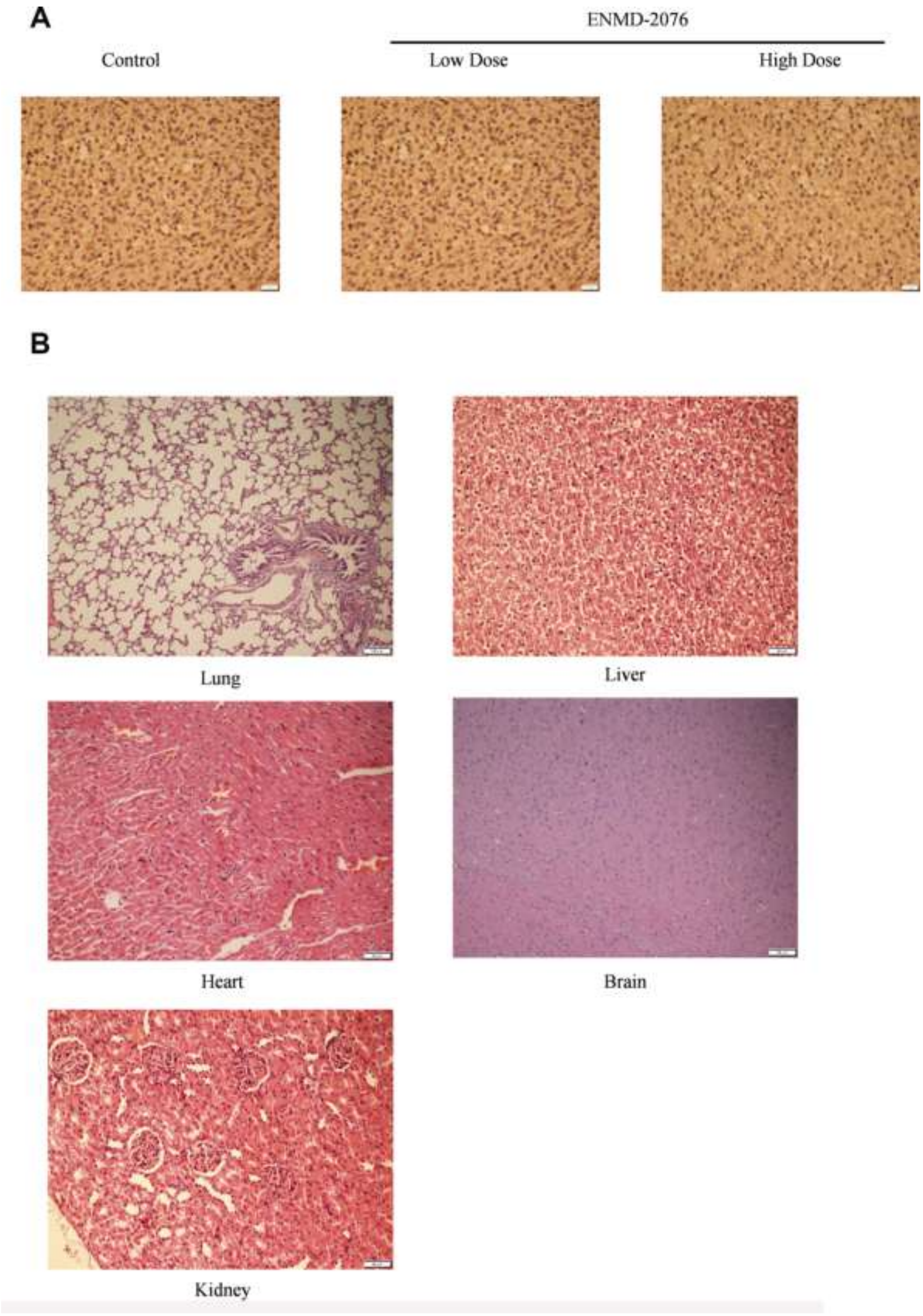

Supplementary Figure 4. (**A**) Immunohistochemistry analysis of ENMD-2076 in the different groups. (**B**) Histological figures of the brains, lungs, livers, kidneys and hearts of the rats treated with high doze ENMD-2076.
