# Supplementary material for: Selected by gene co-expression network and molecular docking analyses, ENMD-2076 is highly effective in glioblastoma-bearing rats
Source: Aging (Albany NY). 2019 Nov 9;11(21):9738–66. doi: 10.18632/aging.102422 (PMC6874459; doi:10.18632/aging.102422)
Supplement: Supplementary Tables [file aging-11-102422-s001.pdf]

SUPPLEMENTARY TABLES

Supplementary Table 1. Detailed information of the hub genes in blue module.

Gene symbol	Degree	Betweenness	Gene symbol	Degree	Betweenness
TOP2A	79	0.159972	KIF23	57	0.008236
CDK1	65	0.038539	RRM2	57	0.018541
BIRC5	62	0.034136	FOXM1	57	0.023411
NDC80	60	0.024477	AURKA	56	0.005829
TTK	59	0.010768	CCNB2	56	0.013458
BUB1B	58	0.016069	AURKB	56	0.006843
DLGAP5	58	0.008631	CEP55	56	0.01069
CDC20	57	0.018397	MELK	56	0.007731
BUB1	57	6.82E-04	CHEK1	55	0.007047
KIF11	57	0.003164	PBK	55	0.007265

Supplementary Table 2. Functional and pathway enrichment analysis of MCODE identified genes.

Module	Term	Count	P Value	FDR	Genes
1	GO:0000279~M phase	28	2.44E-32	3.41E-29	KIF23, TTK, CHEK1, AURKA, CEP55, AURKB, KIF2C, OIP5, BUB1, ASPM, CDK1, KIF11, MKI67, DLGAP5, KIF15, NUF2, TPX2, CENPF, NUSAP1, ESPL1, BIRC5, CENPE, NDC80, CDC20, PBK, UBE2C, CCNB2, BUB1B
	GO:0000280~nuclear division	25	1.86E-31	2.59E-28	KIF23, CDK1, KIF11, DLGAP5, KIF15, TPX2, NUF2, CENPF, NUSAP1, ESPL1, AURKA, NDC80, CDC20, CENPE, BIRC5, AURKB, PBK, CEP55, UBE2C, KIF2C, CCNB2, OIP5, BUB1, BUB1B, ASPM
	GO:0007067~mitosis	25	1.86E-31	2.59E-28	KIF23, CDK1, KIF11, DLGAP5, KIF15, TPX2, NUF2, CENPF, NUSAP1, ESPL1, AURKA, NDC80, CDC20, CENPE, BIRC5, AURKB, PBK, CEP55, UBE2C, KIF2C, CCNB2, OIP5, BUB1, BUB1B, ASPM
2	GO:0004890~GABA-A receptor activity	9	2.17E-17	2.54E-14	GABRD, GABRG1, GABRG2, GABRA2, GABRA1, GABRA4, GABRB2, GABRB1, GABRA5
	GO:0016917~GABA receptor activity	9	2.10E-16	2.55E-13	GABRD, GABRG1, GABRG2, GABRA2, GABRA1, GABRA4, GABRB2, GABRB1, GABRA5
	GO:0034707~chloride	10	3.37E-15	3.59E-12	GABRD, GABRG1, FXYD1, GABRG2, GABRA2, GABRA1, GABRA4, GABRB2, GABRB1, GABRA5

Supplementary Table 3. ADMET (Adsorption, Distribution, Metabolism and Excretion) and toxicity properties of compounds.

Number	Compounds	BBB level[a]	Absorption[b]	Solubility[c]	Hepatotoxicity[d]	CYP2D6[e]	PPB Level[f]
1	ZINC03918087	4	3	2	1	0	0
2	ZINC58661275	3	0	2	0	1	0
3	ZINC06718723	3	0	3	0	0	1
4	ZINC34885047	2	0	3	0	1	0
5	ZINC15449300	4	3	2	1	0	0
6	ZINC34842284	4	0	3	1	0	2
7	ZINC13643145	3	0	3	0	0	2
8	ZINC28824607	3	0	2	0	1	0
9	ZINC14951647	3	0	3	0	0	1
10	ZINC28714095	4	1	2	1	1	2
11	ZINC03814434	2	0	1	1	1	2
12	ZINC43129461	4	3	3	1	0	0
13	ZINC03939511	4	0	1	1	0	1
14	ZINC72178031	4	0	3	0	0	2
15	ZINC06718665	3	0	3	0	0	2
16	ZINC38995988	3	0	3	0	0	0
17	ZINC16697102	4	0	3	1	0	0
18	ZINC35880991	4	2	1	1	0	2
19	ZINC00988000	4	1	1	1	1	2
20	ZINC58660958	3	0	3	0	0	0

[a]Blood Brain Barrier level: 0 (Very high penetrant); 1 (High); 2 (Medium); 3 (Low); 4 (Undefined); [b]Human intestinal absorption level: 0 (good); 1 (moderate); 2 (poor); 3 (very poor); [c]Aqueous solubility level: 0 (extremely low); 1 (very low, but possible); 2 (low); 3 (good); [d]Hepatotoxicity: 0 (Nontoxic); 1 (Toxic); [e]Cytochrome P450 2D6 level: 0 (Non-inhibitor); 1 (Inhibitor); [f]Plasma Protein Binding: 0 (Binding is <90%); 1 (Binding is>90%); 2 (Binding is >95%).

Toxicity properties of compounds

Number	Compounds	Mouse NTP[a]		Rat NTP[a]		AMES[b]	DTP[c]
		Female	Male	Female	Male		
1	ZINC03918087	0	1	1	1	0	1
2	ZINC58661275	0	1	0	0	1	0
3	ZINC06718723	1	0	0.067	1	0.998	0.965
4	ZINC34885047	0	0	0	0	1	0
5	ZINC15449300	0	1	1	1	0	1
6	ZINC34842284	1	1	0.999	1	1	1
7	ZINC13643145	0.501	0	0	1	1	0.732
8	ZINC28824607	0.001	0.995	0	0.177	0	0
9	ZINC14951647	1	0	0.067	1	0.998	0.965
10	ZINC28714095	1	0	0.779	0.514	0	1
11	ZINC03814434	1	0	0.003	0.543	0	1
12	ZINC43129461	1	1	0	0.628	1	0
13	ZINC03939511	1	1	1	0.973	1	1
14	ZINC72178031	0.61	0	0	0	1	0
15	ZINC06718665	0.002	0	0	1	1	1
16	ZINC38995988	0.474	1	0.169	0	0	0.999
17	ZINC16697102	1	1	0	0.03	1	0
18	ZINC35880991	1	1	0	0	0	1
19	ZINC00988000	0	0	0	0	0	0.983
20	ZINC58660958	1	1	0	0	0.997	0.001

[a]0 (Non-Carcinogen); 1 (Carcinogen); [b]0 (Non-Mutagen); 1 (Mutagen); [c]0 (Non-Toxic); 1 (Toxic).

Supplementary Table 4. Hydrogen bond interaction and Pi interaction parameters for each compound and Aurora A kinase and VEGFR residues.

Receptor	Compound	Donor Atom	Receptor Atom	Distance (Å)
Aurora kinase A	ENMD	ASN261:OD1	ENMD-2076:H53	1.92
		GLU211:O	ENMD-2076:H51	2.59
VEGFR-2	ENMD	ASP1046:O	ENMD-2076:H54	3.08
		ASP1046:O	ENMD-2076:H48	2.93

Pi interaction parameters for each ligand-receptor complex

Receptor	Compound	Pi-Cation		Pi-Sigma		Pi-Alkyl		Distances (Å)
		Donor Atom	Receptor	Donor Atom	Receptor	Donor Atom	Receptor	
Aurora kinase A	ENMD-2076	ASP274:OD2	ENMD-2076:H53	NA		NA		2.00
		ARG220:NH1	ENMD-2076					3.62
		ARG137:NH2	ENMD-2076					4.88
VEGFR-2	ENMD-2076	NA		VAL898:CG1	ENMD-2076			3.81
						VAL899	ENMD-2076	5.20
						VAL916	ENMD-2076	5.35
						LEU1019	ENMD-2076	4.72
						LEU889	ENMD-2076	4.85

NA notes not available.
